# Co-design workshops to develop evidence synthesis summary formats for use by clinical guideline development groups

**DOI:** 10.1186/s13643-024-02518-z

**Published:** 2024-03-27

**Authors:** Ruairi Murray, Erindaa Magendran, Neya Chander, Rosarie Lynch, Michelle O’Neill, Declan Devane, Susan M. Smith, Kamal Mahtani, Máirín Ryan, Barbara Clyne, Melissa K. Sharp

**Affiliations:** 1 Health Information and Quality Authority, Dublin, Ireland; 2 School of Medicine, RCSI University of Medicine and Health Sciences, 123 St. Stephens Green, Dublin, Ireland; 3 Department of Health, National Patient Safety Office, Dublin, Ireland; 4 School of Nursing and Midwifery, University of Galway, Galway, Ireland; 5 Evidence Synthesis Ireland & Cochrane Ireland, University of Galway, Galway, Ireland; 6 Discipline of Public Health and Primary Care, School of Medicine, Trinity College Dublin, Dublin, Ireland; 7 Department of Primary Care Health Sciences, University of Oxford, Oxford, England; 8 Department of Pharmacology & Therapeutics, Trinity Health Sciences, Trinity College Dublin, Dublin, Ireland; 9 Department of Public Health and Epidemiology, School of Population Health, RCSI University of Medicine and Health Sciences, 123 St. Stephens Green, Dublin, Ireland

**Keywords:** Clinical guidelines, Evidence summaries, Evidence synthesis, Summary formats, Qualitative research, Focus groups

## Abstract

**Background:**

Evidence synthesis is used by decision-makers in various ways, such as developing evidence-based recommendations for clinical guidelines. Clinical guideline development groups (GDGs) typically discuss evidence synthesis findings in a multidisciplinary group, including patients, healthcare providers, policymakers, etc. A recent mixed methods systematic review (MMSR) identified no gold standard format for optimally presenting evidence synthesis findings to these groups. However, it provided 94 recommendations to help produce more effective summary formats for general evidence syntheses (e.g., systematic reviews). To refine the MMSR recommendations to create more actionable guidance for summary producers, we aimed to explore these 94 recommendations with participants involved in evidence synthesis and guideline development.

**Methods:**

We conducted a descriptive qualitative study using online focus group workshops in February and March 2023. These groups used a participatory co-design approach with interactive voting activities to identify preferences for a summary format's essential content and style. We created a topic guide focused on recommendations from the MMSR with mixed methods support, ≥ 3 supporting studies, and those prioritized by an expert advisory group via a pragmatic prioritization exercise using the MoSCoW method (Must, Should, Could, and Will not haves). Eligible participants must be/have been involved in GDGs and/or evidence synthesis. Groups were recorded and transcribed. Two independent researchers analyzed transcripts using directed content analysis with 94 pre-defined codes from the MMSR.

**Results:**

Thirty individuals participated in six focus groups. We coded 79 of the 94 pre-defined codes. Participants suggested a “less is more” structured approach that minimizes methodological steps and statistical data, promoting accessibility to all audiences by judicious use of links to further information in the full report. They emphasized concise, consistently presented formats that highlight key messages, flag readers to indicators of trust in the producers (i.e., logos, websites, and conflict of interest statements), and highlight the certainty of evidence (without extenuating details).

**Conclusions:**

This study identified guidance based on the preferences of guideline developers and evidence synthesis producers about the format of evidence synthesis summaries to support decision-making. The next steps involve developing and user-testing prototype formats through one-on-one semi-structured interviews to optimize evidence synthesis summaries and support decision-making.

**Supplementary Information:**

The online version contains supplementary material available at 10.1186/s13643-024-02518-z.

## Contributions to literature

- This study explored the preferences of evidence synthesis producers, those who pull together all the available evidence on a specific topic, and those who make decisions about health care and policies based on that evidence (clinical guideline developers).
- We held focus group workshops with these two groups to explore what content, style, and structure they wanted in summaries of evidence synthesis. We created guidance on best practices for creating evidence synthesis summaries.
- Although producers and developers are diverse groups with different knowledge bases and priorities, we found that participant’s roles largely did not impact the preferences expressed.

## Introduction

“Information is a source of learning. But unless it is organized, processed, and available to the right people in a format for decision making, it is a burden, not a benefit.”—William Pollard.

Clinical guidelines are informed by evidence syntheses which identify and combine data across individual studies to answer questions about specific clinical conditions or topics [1]. The syntheses which conventionally inform guidelines are systematic reviews (with or without a meta-analysis). However, the evidence synthesis field has expanded in recent years to include a wide variety of approaches (e.g., network meta-analysis, rapid reviews). These evidence syntheses are generally quite lengthy and tend to be written in technical academic language, often making them difficult to understand and navigate. Organizations and decision-makers are increasingly using evidence synthesis summaries alongside the full technical report to inform policy and practice [1–4], particularly during emergencies such as the COVID-19 pandemic [1, 4].

Decision-making during the guideline development process involves compromises between the evidence base, clinical experience, and other contextual, ethical, and budgetary considerations [5]. Decision-makers and those involved in guideline development groups (GDGs), such as healthcare managers, providers, patients, and researchers, need evidence synthesis summaries to be relevant, comprehensive yet concise, and written in plain language [4, 6]. Evidence synthesis summaries can come in various formats, such as short reports (1–5 pages), summary of findings tables, visual abstracts, infographics, etc. While summaries may be more easily understandable than complete systematic reviews [3, 7], there is no consensus on the most effective way to communicate what works for whom.

We recently conducted a mixed methods systematic review (MMSR) [8, 9] to identify the most effective and acceptable summary format(s) for different end-users involved in clinical GDGs. Our review findings covered a range of types of evidence syntheses, end-users, and summary formats. We identified no clear preferred summary format yet were able to provide a synthesized list of 94 recommendations (Additional file 1) to help produce more effective and acceptable summary formats of general (i.e., systematic review) evidence synthesis findings. These recommendations spanned six thematic areas, including (1) presenting information, (2) tailoring information for end-users, (3) contextualizing information, (4) trust in producers and summary, (5) knowledge required to understand findings, and (6) quality of evidence. To refine the list of MMSR recommendations to create more actionable guidance for summary producers, we aimed to explore these 94 recommendations through focus group workshops with participants involved in evidence synthesis and guideline development. For our purposes, this meant that rather than taking the MMSR recommendations and internally deciding upon which to embrace and use to develop evidence synthesis summary formats, we would instead explore these recommendations with end-users who may use these formats in the future.

## Methods

We pre-registered our protocol on Open Science Framework [10] and are reporting according to the COREQ guideline (Additional file 2) [11]. We obtained ethical approval from the Research Ethics Committee at the RCSI University of Medicine and Health Sciences (RCSI) (REC 202111005).

### Study design

We employed a qualitative design using focus groups and a co-production approach which typically involves researchers sharing responsibility and power with participants to generate knowledge and explore what works for whom and in what context [12–15]. This co-design approach allowed us as researchers to work collaboratively with participants to let them lead us in determining what were the essential design features or recommendations that they wanted in an evidence synthesis summary format. We developed a facilitation and topic guide (Additional file 3) informed by SENSES guidelines for co-production workshops manual [12] and the UK National Institute for Health and Care Research (NIHR) Guide to Co-production for Researchers, Services and Commissioners [13].

#### Research team and reflexivity

The lead facilitator (MKS) is a mixed methods researcher with a background in Psychology (BS) and Epidemiology (MPH, PhD); she also took additional facilitation training. Two co-facilitators (RM, BC) assisted with additional prompts and tech support. The co-facilitator (RM) is a health services researcher working in evidence syntheses (background: Economics and Sociology (BA) and Health Economics (MSc)). Co-facilitator (BC) has extensive experience in evidence synthesis and working with guideline development groups; her background is in Sociology (BSocSc, MSocSc) and Health Services Research (PhD)).

### Workshop development

Our MMSR [8] produced 94 general recommendations (Additional file 1):

- Nine with mixed-methods support (i.e., quantitative *and* qualitative studies)
- Twenty-one supported by at least three individual studies (either qualitative or quantitative)
- Sixty-four with two or less supporting streams of evidence.

To develop a clear yet thorough topic guide for our workshops, we organized sessions around the six key themes outlined in our MMSR. We prioritized discussions based on the strength of evidence supporting each recommendation. Specifically, our focus was on the 30 recommendations that either had mixed-methods support or were backed by at least three individual studies, which we categorized as 'strong support'.

In parallel, we also conducted an online pragmatic prioritization exercise (via Welphi) [16] with our project’s multidisciplinary steering committee (experts in evidence synthesis, clinical care, and guidelines). This exercise helped us identify additional recommendations to integrate as topic guide prompts (Additional file 3). In July 2022, seven steering committee members and one patient representative rated recommendations according to the MoSCoW prioritization method [17], which is used to prioritize the ‘Must Have’, ‘Should have’, ‘Could have’, and ‘Won’t have’ (at this time) requirements for a project or tool (Table 1). The MoSCoW method, commonly used in business, software development, and project management, does not have clear cut-points; thus we focused on the extremes of the scale, areas of discrepancies, and the evidence base as identified in our MMSR [18].

**Table 1:** Summary of MoSCoW prioritization method categories

Must have	Requirements which are essential or critical and must be included for success
Should have	Requirements which are important but not necessary. They add significant value and may be as important as ‘must have’ items but are not as time-critical
Could have	Desirable requirements which can affect user experience or satisfaction and can be included if time and resources allow
Won't have (at this time)	Least-critical, lowest-payback, or not appropriate requirements. They are not a priority and are outside of the scope of delivery at this time

### Participant recruitment

We used purposive sampling to recruit a mix of participants, including patient representatives, decision-makers, healthcare administrators, clinicians, healthcare providers, and methodologists. Co-authors from Ireland’s Department of Health (DoH), the Health Information and Quality Authority (HIQA), and RCSI (RL, MO, MR, MKS) acted as gatekeepers and emailed information leaflets to potential participants. Participants were eligible if they were involved in evidence syntheses informing clinical guidelines or at least one decision-making (e.g., Expert Advisory Group) or clinical guideline development group. Focus group facilitators may have previously collaborated with some participants through work in evidence synthesis or guideline development. Our protocol established a recruitment goal of at least 15 participants across three workshops [10]. Due to the variety of end-users and the number of MMSR recommendations [9], we aimed for six focus groups with at least four to seven participants each. We purposefully arranged groups to be multidisciplinary to avoid ‘group-think,’ aiming for a maximum of seven per group. After six groups, we discussed saturation as a team (BC, RM, MKS) and decided that new groups would not produce value-added insights.

### Workshop format

**Fig. 1:**
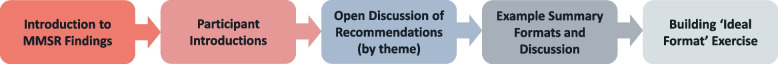
Format of workshop discussions

We invited participants to join a 90-min co-design focus group workshop on Zoom (Fig. 1).

**Fig. 2:**
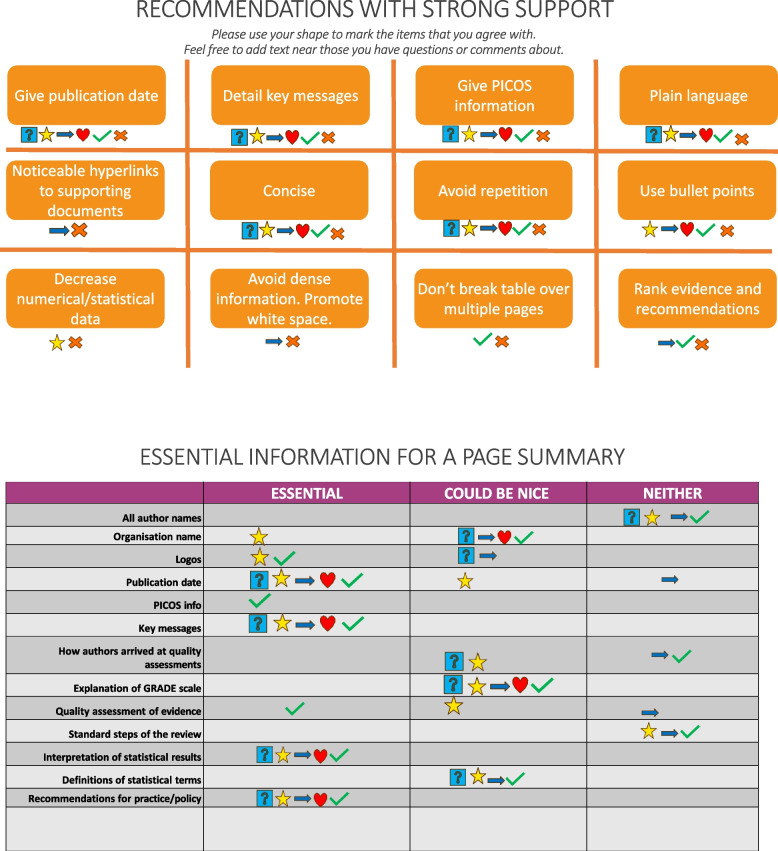
Examples of voting activities

The structure of each was the following:

1. Introduction to MMSR findings and the discussion’s focus on discussing the MMSR recommendations, particularly focusing on written summary formats (i.e., not audiovisual formats).
2. Participants introduced themselves, including their evidence synthesis and guideline development background.
3. PowerPoint presentation of recommendations and discussion by theme and in order of support (strongest to weakest) (Additional files 3 and 4). This facilitated discussion included an interactive Zoom stamping activity to hone in on areas of consensus and disagreement. Participants were assigned individual stamps (e.g., star, heart) before the workshop (Fig. 2).
4. Three example summary formats of different lengths and formats were shown to participants, who then voted on their favorite and discussed what aspects they (did not) like.
5. ‘Building exercise’ where participants openly discussed and voted on a table of items as being ‘essential,’ ‘could be nice (to have), or ‘neither’ to have in a 1-, 3-, or 5-page summary.

Based on discussions and time, we modified which themes were shown (Table 2), aiming for an overall group balance. After all data was coded and summarised, participants were invited to a recorded ‘debriefing’ session where results were presented and participants were asked to give any final feedback on the preliminary results and offered a chance via email to review their transcripts.

**Table 2:**
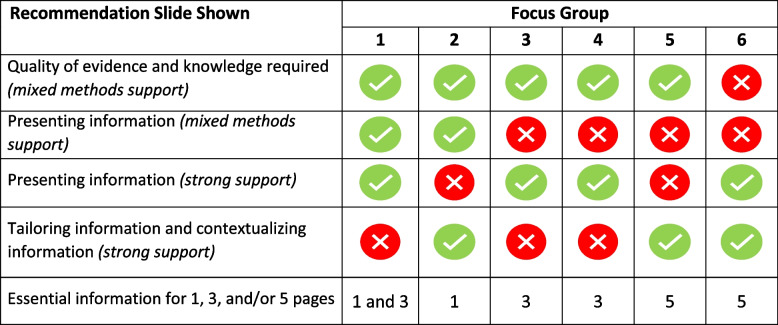
Recommendation slides shown

### Analysis

We video-recorded and transcribed focus groups for a directed content analysis [19] using pre-defined codes stemming from the MMSR. This analysis aims to validate or extend a theoretical framework or theory. It aligns with our data collection approach which used open- and close-ended questions related to the MMSR’s 94 recommendations. Two reviewers (RM, MKS) independently reviewed all transcripts to familiarise themselves with the data and coded half each (evens and odds) in NVivo. Another two reviewers independently double-coded each transcript (EM, NC). Coding was combined and discrepancies were addressed by the lead facilitator (MKS).

We linked codes to the MMSR’s 94 recommendations. Text that could not be coded to one of the predetermined categories (i.e., recommendations) was given a new code. We initially planned on coding participants’ preferences concerning their group membership (i.e., ‘as a clinician, I prefer…) but many individuals were multidisciplinary themselves (e.g., clinician researchers) and nuanced differences were rare. We also coded whether participants did *not* support a recommendation. On-screen activities (Fig. 2) were meant to be conversation-generating, not a quantitative polling exercise. Furthermore, during several discussions, people stated that they had changed their opinions, contradicting their stamp. Regardless, we reviewed responses after textual analysis.

## Results

### Participants

Thirty of 42 who expressed interest in participating were available and consented to take part in a focus group. We had 4–6 people in each group and the majority of participants described their primary role as being academic, researcher, or methodologist, however, many individuals were multidisciplinary themselves (Table 3). For example, we had academics and methodologists who were previously healthcare providers. The majority of participants were familiar with summary formats with most reporting familiarity with abstracts (*n* = 29), plain language summaries (*n* = 26), and summary of findings tables (*n* = 24).

**Table 3:** Participant demographics

Characteristic	*N* = 32^a^
What is your primary role (in relation to clinical guideline development or expert advisory groups)?	
*Patient representative*	1
*Healthcare manager or administrator*	1
*Policy or decision-maker*	4
*Academic, researcher, or methodologist*	17
*Healthcare provider*	4
*Communications or graphic designer*	1
*Other*	4
Age group	
*18–24*	0
*25–34*	12
*35–44*	10
*45–54*	5
*55–64*	5
*65* +	0
What kinds of evidence synthesis summaries have you interacted with? (select all that apply)	
*Policy briefs*	11
*1, 3, or 5 page summaries*	22
*Abstracts*	29
*Summary of findings tables*	24
*Plain language summaries*	26
*Infographics or visual summaries*	22
*Podcasts*	3
*Video summaries*	3
*Other*	3
Which evidence synthesis summaries do you prefer? (select all that apply)	
*Policy briefs*	8
*1, 3, or 5 page summaries*	15
*Abstracts*	4
*Summary of findings tables*	14
*Plain language summaries*	13
*Infographics or visual summaries*	19
*Podcasts*	1
*Video summaries*	1
*Other*	0

^a^To reduce collection of personally identifiable information, survey data was not tracked and due to last minute drop-outs, only 30 participants ended up joining the co-design workshops

We coded to 79 of the 94 recommendations and generated three new codes relating to describing caveats, the ability to navigate, and color preference. These 79 recommendations were categorized into structure, style, and content for ease of presentation. Of note, the 15 recommendations that we did not code to were largely only from one supporting study, often dealt with minute details (e.g., ‘shade rows’, ‘present positive results first, then negative’) or perhaps may not have been deemed relevant for our participant population (i.e., all participants were native-English speakers and did not discuss ‘use end-user’s native language’). The 15 recommendations are available in Additional file 1 with red strikethrough text. In addition to text being coded to the recommendation itself, text was coded as ‘Not Supporting’ to indicate if a participant objected or did not agree with a recommendation. At most, this was 7% of the coding coverage in any of the 6 transcripts, and special attention was given to these codes to present a balanced presentation of the results below.

### Structure and style

Although summaries of varying lengths were discussed, including executive summaries of nearly ten pages, most participants agreed that *“*whether it is a policy brief or a plain language summary or an executive summary…” 1–2 pages was preferred over “…a five pager or six pager” [039]. One-page summaries were preferred by most. It was recognized that “it takes such effort to get things simplified and readable but it’s so worth it” [033] but it has its benefits as “sometimes the more you write the more you have to explain…” [027].

Participants also generally agreed that a visual format may be more helpful as “visual representation can get the message across very powerfully” [022] although it was recognized that some topics may be easier to present visually: “this thing was an easy thing to put an image of, which you can’t do with everything” [012]. While a format that balances visual and textual information was desired by many, concerns about resource constraints were expressed with one participant reporting “that the biggest barrier is for people to actually be able to produce them, I'd love to be able to do it if I had the software and the skills and the time” [027].

Even if the resources are available, participants did not like overly illustrated approaches as this was seen as largely inaccessible, with one participant noting that “there’s a lot of time given to…making things…visually appealing for some people that will actually limit it for other people…high level summaries…should be accessible to everyone” [028]. Aspects of accessibility and readability that several participants commented on were the “real balance” [037] of text and white space, bullet points, subheadings, easily identifiable hyperlinks, and simple color schemes which emphasized important information such as key messages. Most participants agreed that key messages were one of the most important aspects of a summary, stating: “the focus in a summary is more on the results than the methods” [027]. There was no agreement on whether the key message should be presented first or last in a summary but as one participant noted, “if it's high level enough and if you have done it right, you should want people to work through everything before they get to…the key messages” [037]. Hyperlinks “inserted in all the places where they’re relevant as opposed to having at the end tools and resources section” were praised by many.

Flexibility in the presentation of information was discussed (e.g., collapsible sections with interactive formats) but as one participant expressed: “it would be nice to have that but also you’d have to make sure that there’s some level of control that people are getting the key message still, they’re not just picking and choosing pieces that they want to see” [032]. Flexibility was also discussed in relation to reading preferences for printed versus online summaries. Some preferred printable formats as they “just find it easier to digest something that’s actually on a piece of paper in front of me as opposed to on a screen” [020]. Even for those who “prints the 80 page report. And reads it and highlights it and you know underlines…,” it was acceptable to have “the one open on the laptop and clicking the hyperlink…” [012]. Hyperlinks were also generally preferred to footnotes as they’re “…more immediate. A footnote takes up text and a link, it's optional whether you go to it or not and it's immediate for the reader” [011].

Regarding subheadings and structure, most participants did not like the IMRaD (Introduction, Methods, Results, and Discussion) academic format, which has been the predominant structure used in academic articles since the 1970s [20]. For synthesis summaries, they expressed that “it’s more user friendly for everyone involved” [012] not to use it (IMRaD). They liked smaller structured sections that “have it broken up” [037] with signposting to important information, stating that “it can be nicer to read a shorter piece and then go on to the next…as opposed to being…presented with a huge block of text that’s a bit overwhelming" [012]. These discussions related to the expressed need to clarify the audience for the evidence summary. Accessibility for all end-users was emphasized over individual separate tailored summaries: “you are never going to get something that suits everybody and someone is going to want more information. But if you have appropriate links to that other information I think a one size fits all could work” [015].

Accessibility for all end-users was also related to the need to consistently format summaries over time. It was recognized that “each organisation will have their own format” [024] but that “consistency in the format is really important it's…marketing so you have to…be…consistent because I think people get used to using a certain thing and they get familiar with it and they like it” [009]. In addition to being helpful from an organizational point-of-view, decision-makers also viewed this consistency as “great…it gave me an idea of…the direction of flow or…what needed to be completed” [001] and it helps end-users “know exactly where to find stuff” [016].

### Content

This consistency from organizations or summary producers was also related to participant’s trust in the summary findings, with some expressing that “there needs to be clear ownership of where…” a summary “…has come from because people will be copying and pasting it…” One participant noted that “you’d have confidence in certain institutions…that the assessment was rigorous” [010] with another echoing that “I think the reputation of the organisation is very important…” [024] Recognizable logos and links to organization websites or the first author et al. were preferred over a full list of authors. Disclosures of “some sort of funding and conflict of interest” [040] were also deemed important even if it was a simple note of “no conflicts of interest or conflicts are reported, go here for information. You don’t need every single thing on the summary itself but an indication that there are or not” [040]. This was “important, particularly for pharmaceutical policies or medicine policies” [024].

Other important information to signpost included key messages and the need to communicate “why there is a summary of the evidence” [014] and properly framing the context by describing the PICO (patients, interventions, comparators, and outcomes) as it “tells you everything you need to know about…who it is you’re talking about, what it is you’re talking about and in terms of intervention and…what outcomes you’re interested in” [034]. It was noted that defining the scope of the question being addressed could be particularly helpful when a “guideline group will discuss something that’s not there and they’ll say well what about this paper. And you have to go well you didn’t ask that question” [034]. PICO information was deemed essential to include with a strong preference for a narrative format, i.e., “covered in terms of the introduction” [004], or a “smart art graphic as opposed to an absolute table” [024]. There was some disagreement about whether participants (did not) like a P-I-C-O bulleted presentation. The context or scope of the findings was emphasized as the “inclusion criteria and exclusion criteria” were essential to “judge the results in terms of how something has been done…” [015].

Additional important contextual information was the synthesis's search and/or publication date. However, caution was expressed that putting both may confuse readers, as one participant stated, "I would automatically assume that I was wrong, if I saw something that was like March 2020 but they just published it March 2022. I’d think maybe there was something wrong” [014]. Those involved in synthesizing the evidence noted that “they take quite a while between searching and publishing, I think the search date would be more useful than publication date” [008] highlighting the importance of knowing the recency of the evidence base—“especially in something like COVID where there’s very rapidly developing evidence. You’d want to know how far back does this go into” [032]. Ultimately, “whether or not that includes the dates or not, I guess it depends on how relevant that might be for the key message” [030].

### Methodology

**Fig. 3:**
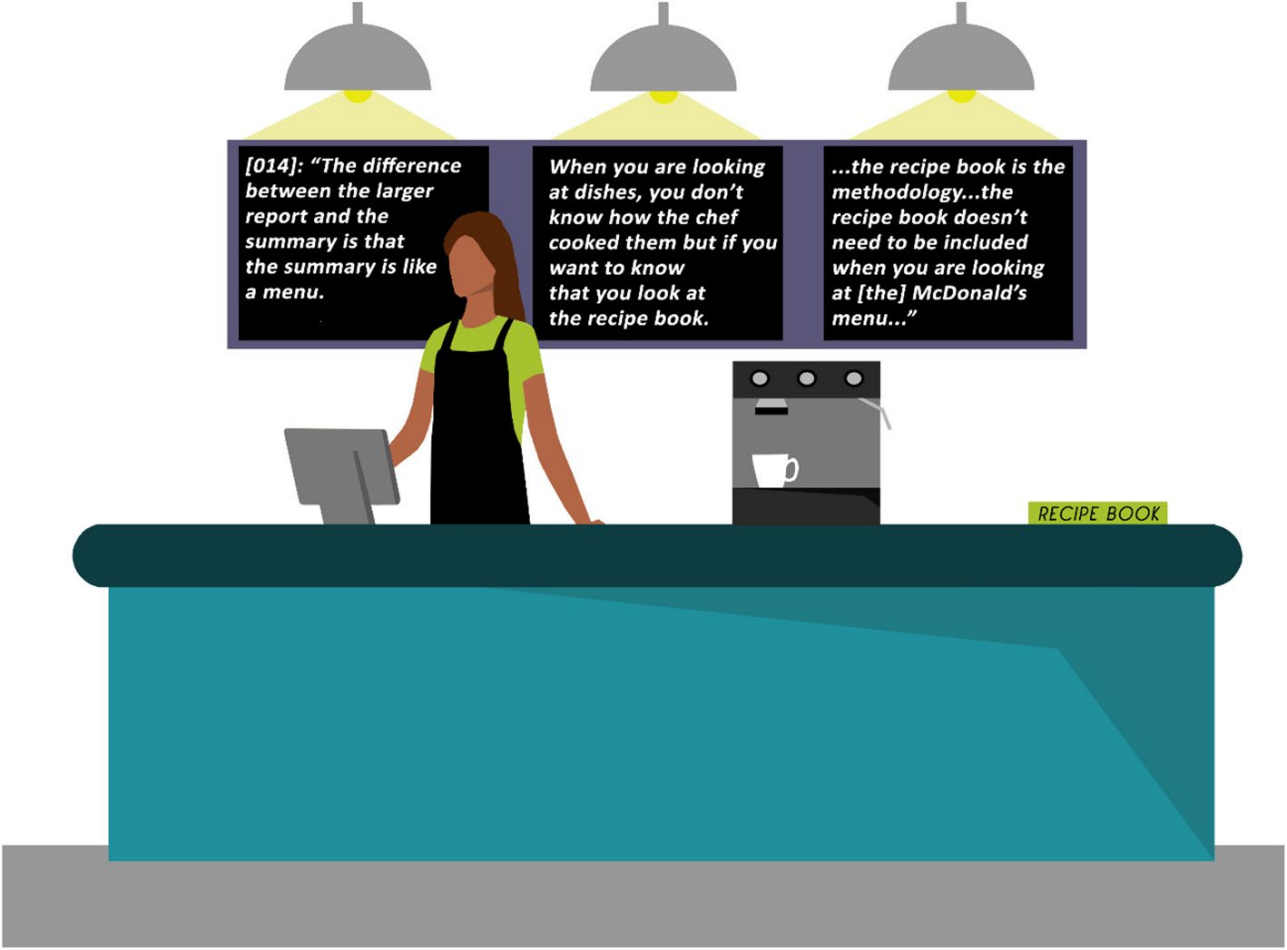
Example quote

Aside from the search and publication dates, there was much discussion about the evidence synthesis methodology (Fig. 3). It was broadly agreed that important information such as the type of review, “like this was a rapid review as opposed to…a systematic review…” [032] should be included but “…we’re not talking about the steps of a systematic review” [032]. A majority of participants, even methodologists,

“don’t think detailed methods work belongs in the summary…We know where to go if we want to find anything to do with methods and it doesn’t go into the summary, people know to go to the report for that…I don’t even think it's a nice to have, I think it can confuse a reader as to the point of the report. And it might indicate that what they’re reading is not for them.” [042]

Participants agreed that the “methods absolutely have to be reported for transparency” but that information generally should be in “an appendix where if people wanted to…review it in that level of detail,” [018] they could. Framing methods “a bit differently, like what did we do?” [027] was viewed as more accessible as “it's already more conversational and easier to understand than talking about methodology and…that is jargon at the end of the day” [027]. Most participants agreed that the details such as which assessment tools were used, would be within the full technical report, not the summary: “if people wanted to know more about what the steps were in that review then again I would…maybe put in a link and they can review that those steps have been taken” [016].

### Statistical information

The idea that “bombarding them with methodology probably isn’t the best way to go” [027] extended to providing definitions of statistical terms which was also seen as a “waste of your word count” [027], particularly for clinicians who “want the bigger picture and what impact that’s going to have on their clinical practice” [020]. However, it was noted that it was “important to have those definitions in there…” for things that could be “…easily misinterpreted by an unfamiliar reader” [028]. Interpreting statistical findings was emphasized over actual numerical statistics; it was “better not to use statistical terms or even any statistical association or like odds ratio…” [031] and that it is more helpful to add “a contextualization part to add a bit of meaning behind the statistic…” meaning “…what is the result saying in terms of the finding that you are talking about. So is it going to lead to an increased risk, a decreased risk” [026].

If possible “statistics…in diagram form” [011] such as “stick people…coloured in to show numbers, figures, and things like that” [035] were helpful because “if you have a big paragraph that’s just giving you statistical information with R values, that’s numbing. While if you can pull it out and actually showcase it through like imagery…it can be seen in a context” [014]. Exceptions to minimizing numerical information were discussed such as when “it's more nuanced or more borderline” [040] or when “it's like a major thing that’s going to really impact the guideline or impact or practice…it can be helpful just to have like the actual P value or confidence interval there…sometimes to see like how wide is that interval or what are we actually dealing with here…” [030]. Yet still, minimizing information was preferred: “you don’t want a high-level summary just to be full of like P values or some other tests” [030].

Presenting statistical information for guideline development can often come in the form of a summary of findings tables. These were discussed as “very important for guideline development groups” [027] with some stating that “definitely summary finding tables should be added.” [031] However, it was noted that they can be “overwhelming…” particularly when there are.

“…multiple time points, multiple outcomes…” because “…then the reader is kind of left wondering well which is the most important time point, which is the most important outcome?” [027] It was suggested that one could “break up the summary tables findings…into categories or something like that. So that it’s not one big, long table…” [015].

### Certainty of evidence

The importance of including an assessment of the quality of evidence (largely in reference to the Grading of Recommendations Assessment, Development, and Evaluation or GRADE scale) in an evidence summary was discussed at length. Many felt that, in addition to the key messages, “the most important thing is the person would walk away from reading your summary and say it's either high or low quality” [027]. Some participants strongly felt that providing “information on the GRADEing of the evidence” was “how we can generate the trust on the evidence” [031]. But others cautioned that while “GRADE is obviously an ideal…sometimes though it can be misinterpreted so if something is very low certainty evidence it doesn’t necessarily mean it doesn’t work or is bad” [025]. Others echoed the concern with using and the need to explain GRADE, expressing that they “think the GRADE is way too deep. That scale doesn’t need to be explained at all.” [014] Participants agreed that if GRADE is used it should be a “generic overview” [028] or explained “in a very easy way or in an explainable way. Not using the jargon” [031].

### Recommendations

There was some disagreement regarding putting recommendations in an evidence summary. Some thought, "if a guideline has been commissioned to inform policy…it's an opportunity not to leave that unsaid” [011]. As one participant framed it, if a synthesis was from a governmental statutory body or clinical program for a “specific context then it’s appropriate. So it totally depends on where you sit within that decision making context” [027]. However, another participant preferred a more cautious presentation, noting that “in Cochrane reviews they provide something called author’s conclusions…it is clearly written that it is the authors opinion based on the summary or based on the evidence” [031]. They expressed the belief that “we are providing the evidence so our task is not to recommend anything. Our task is to present the result and to present the…quality of evidence…whether this will be converted into a guideline or not so that is the decision for the decision makers” [031]. However, “from a clinicians’ point of view…the recommendations are the most important and…how they can be implemented in the clinical setting” [005]. As these evidence summaries are informing clinical guidelines, one patient frankly stated “that what they need is to know what to do…there isn’t time for fluffing and faffing” [038].

Framing findings, whether recommendations or conclusions, “within the country or the system you’re working in” [028] was noted as a “critical piece” [030] to report in a summary. This information is helpful for any guideline development group members who may be involved in an implementation with some saying “to me implementation is quite crucial because you can say here’s the recommendations, here’s the key points. But you need to figure out how to actually implement it” [032].

### Guidance for summary producers

**Fig. 4:**
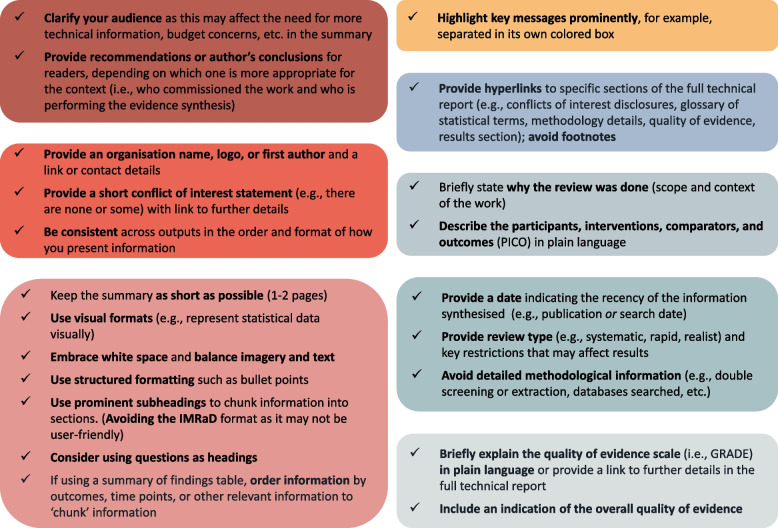
Guidance for summary producers

As one participant noted, “there isn’t really anything… for a general evidence summary that isn’t targeted at a lay audience” [027], thus we aimed to summarise the results from our direct content analysis in a visual (Fig. 4) and one-page-summary (Additional file 5) format to help provide clear and accessible guidance for summary producers. All participants were presented with a draft version of Fig. 4 via email and invited to give comments and attend a debriefing session where the lead facilitator (MKS) explained each item more thoroughly and summarised the results across the six groups. Participants requested minor clarifications and text editing but there were no objections to the content of the guidance.

## Discussion

Our focus group workshops allowed us to create a more practical list of recommendations that was co-designed with multidisciplinary clinical guideline development group members who use and create evidence synthesis summaries for decision-making. Our study’s findings largely confirmed the 94 MMSR recommendations [8] and condensed them into more practical guidance containing 21 items. The guidance for summary producers generated from this research, emphasizes a “less-is-more” approach that avoids academic technical language, limits methodological information, and highlights the key conclusions or recommendations framed within the appropriate context. For all end-users, but particularly clinicians, the key message and recommendations (or conclusions, based on who commissioned the evidence synthesis), were essential to include along with an overview of the quality of the evidence synthesized.

Our guidance aligns with recent work on evidence summaries in health and humanitarian crisis situations, echoing the need to clarify the target audience, presenting key findings in a structured, concise, and visual way, provide implementation considerations, the quality of evidence, and the hyperlinks to the full-text reviews [4]. Our project’s findings recommend evidence synthesis summaries which minimise methodological details. This is also supported by previous research with end-users of systematic reviews [21] which found that unfamiliar methods and terminology were barriers to fully understanding findings. A brief mention of methods, emphasizing key messages, and avoiding GRADE terminology (i.e., ‘low-certainty evidence’) is also promoted by Cochrane’s guidance for plain language summaries (PLS) [22]. Echoing one of our participants’ comments about “how there isn’t really anything…for a general evidence summary that isn’t targeted at a lay audience,” it should be noted and emphasized that the audience for a PLS is the general public and Cochrane PLS are generally intended as summaries of Cochrane Reviews, not for use in the complex setting of decision-making for healthcare policies and practice. In the guideline development context, questions can often be broader and more complex and may not fit with the conventional systematic review model of effectiveness. While our work is also aligned with Cochrane’s Checklist and Guidance for dissemination findings from Cochrane intervention reviews, their guidance states that the dissemination products they aim to improve are focused ‘presenting the findings from Cochrane intervention reviews (i.e., of effectiveness)’ [23].

We found that participants deemed discussing the quality of evidence essential; however, they often believed that the GRADE rating should only be provided in a very easily explainable way. Santesso et al.’s list of informative statements to communicate the results of systematic reviews provides suggested statements to indicate to readers the size of the effect estimates in the context of high, moderate, low, and very low certainty evidence [24]. For example, for a large effect with high, moderate, and low certainty evidence one can make minor changes to reflect this, stating that ‘X results in…’, ‘likely results in’, or ‘may result in…’ ‘a large reduction/increase in the outcome.’ These statements are already being used in practice as they are currently automatically generated in the GRADEpro software [25].

Although there was overall agreement with the MMSR recommendations [9], one area of debate was around the use of recommendations or ‘author’s conclusions.’ Cochrane’s guidance for plain language summaries does not advocate for providing recommendations [22]. However, in the clinical guideline development process, some participants, particularly the clinicians and patient representative, expressed that recommendations were appropriate, with other methodologists supporting this view based on who has commissioned the work (i.e., a national body).

Contextual information that would help with the implementation of changes to healthcare and policies was discussed as essential by participants. Tools such as the CONtextual SENsitivity in SYStematic Reviews (CONSENSYS) instrument may be helpful for summary producers to ensure that their evidence summaries consider context-specific dimensions needed for their audience. This can help guideline groups more effectively translate evidence into their local settings [26]. Tailored evidence resources may aid in more effective decision-making around health policy and practice [4] but our results argue that judicious use of hyperlinks to relevant areas of the full technical report may be sufficient to address more nuanced individual preferences.

Some research [27–29], including our MMSR [9], posited that a one-size-fits-all format may not be feasible due to individual differences in knowledge bases and priorities from the multidisciplinary members in clinical guideline development groups. In fact, we critiqued in our MMSR that the included qualitative studies often did not indicate a person’s role (e.g., clinician, patient, decision-maker) when reporting participant quotes. However, we found that a participant’s role did not have a large impact on preferences expressed, for example, methodologists did not believe that their methodological and statistical information belonged in a summary and were content with hyperlinks to relevant section(s) in the full technical report. Furthermore, many of our participants were multidisciplinary themselves (e.g., former researchers who are currently healthcare providers); this may have also been the case in the studies included in the MMSR. This multidisciplinary nature may have also contributed to the ‘for the good of the group’ mentality.

### Limitations

The goal of our project is to influence practices across Ireland at a national scale. Therefore, we primarily enlisted participants from Irish networks, including healthcare administrators and patient representatives. This approach may limit the international generalizability of our findings, although it is worth noting that the processes for Clinical Guidelines in Ireland share similarities with those in other countries. Despite concerted efforts to involve a broader patient perspective, we included only one patient representative in our study. Our participants were also largely between 25–44 years of age and preferences may vary more widely among younger and older audiences. Future research should seek a more geographically diverse participant pool across a wider age range and include more patient-representative perspectives. However, findings from studies in other contexts and those focused on communicating to the public [4, 22, 23] bolster our guidance, adding to the robustness of our findings.

The directed content analysis also has inherent limitations in that it may have a strong bias toward finding support *for* a theory rather than not. To reduce bias, we used an audit trail and process (i.e., co-author and peer review of the recommendations aka the predetermined coding categories) throughout the creation of MMSR recommendations. We also allowed for the creation of new codes and created a code for text that indicated that a participant was ‘not supporting’ a recommendation so we could easily hone in on this information. All coding was also independently coded by two coders. Lastly, our findings are restricted to ‘general’ systematic reviews (i.e., systematic reviews of effectiveness); there may be different needs for other types or methods of evidence synthesis such as network meta-analyses, diagnostic test accuracy reviews, rapid reviews, or updates to reviews.

### Implications

We have made our guidance accessible to evidence synthesis producers and users through Fig. 4 and a comprehensive checklist (Additional file 5). Having conducted focus group workshops, we formulated a pragmatic list of recommendations. This list has been co-created in collaboration with members of multidisciplinary clinical guideline development groups, who are well-versed in using and creating evidence synthesis summaries to aid decision-making. Our practical guidance of 21 items aligns with and has streamlined the 94 MMSR recommendations [8]. As we move forward, we intend to develop and user-test the prototype templates through detailed one-on-one semi-structured interviews. Once finalized, our templates and guidance will be readily available to support Ireland's National Clinical Guideline development process and bolster guideline development efforts internationally.

## Conclusions

By engaging with 30 participants over six focus groups, our study offers a comprehensive insight into the desired format for evidence synthesis summaries. The participants, who are deeply involved in GDGs and evidence synthesis, consistently highlighted the value of brevity and clarity. They proposed a structured approach that simplifies methodological and statistical information, maintains a clear and consistent format, and highlights key messages prominently. To foster trust, participants underscored the importance of easily identifiable trust indicators, such as logos, dedicated websites, and transparent conflict of interest statements. Furthermore, they highlighted the need to present the quality of evidence clearly without delving into unnecessary complexities. Building on these findings, our next phase will be centered on developing and user-testing prototype summary formats, harnessing the preferences identified, and aiming to refine evidence synthesis summaries to better support decision-making processes.

### Supplementary Information

**Additional file 1. **94 Recommendations from our Mixed Methods Systematic Review.

**Additional file 2. **COnsolidated criteria for REporting Qualitative Research (COREQ))Checklist.

**Additional file 3. **Topic Guide.

**Additional file 4. **Workshop Slideset.

**Additional file 5. **Guidance for Summary Producers.

## Data Availability

The protocol for this study was previously preregistered on the Open Science Framework (OSF) [10]. Participants did not consent to sharing transcripts publicly and NVivo is a proprietary software therefore data sharing is not applicable to this article. Additional information is available on OSF.
