# Supplementary material for: Co-design workshops to develop evidence synthesis summary formats for use by clinical guideline development groups
Source: Syst Rev. 2024 Mar 27;13:97. doi: 10.1186/s13643-024-02518-z (PMC10967093; doi:10.1186/s13643-024-02518-z)
Supplement: Supplementary file 1 — Additional file 1. 94 Recommendations from our Mixed Methods Systematic Review. [file 13643_2024_2518_MOESM1_ESM.docx]

**Appendix 1.** 94 recommendations from our mixed methods systematic review

| **Key**  * Supported by both qualitative and quantitative evidence  Specifically expressed by clinicians^a^, policy/decision makers^b^, healthcare managers^c^, content experts/academics^d^, guideline committees^e^, patient representatives^f^  *For studies reporting on the same trial (i.e., Hartling 2016 and Hartling 2018* [1] *and Smith 2019 and Totten 2019* [2]*, only one paper is cited as to not visually misrepresent recommendations as being supported by more studies than it is)* |
| --- |

| **Presenting Information** |
| --- |
| **Content**  First page   1. List authors [5,9] 2. Give publication date [5,7,9] 3. Detail key messages [1,5,9,10,12] separated by subheadings [5] 4. Describe relevance for practice/policies [9]^b^   General   1. Plain language [1,4–6,12,16] and jargon-free [7] 2. Avoid repetition [5,6,8] 3. Avoid abbreviations [12]^b^; if necessary, don’t define in footnotes [1]^b^ 4. Consider framing title as question [12]^b^ 5. Prominent subheadings [7] 6. Provide background [5] 7. Give PICOS information [6,15,16] and characteristics of included studies [13] 8. ~~Report what worked and what didn’t [7]~~ 9. Use ‘explanations’ or ‘clarifications’ instead of ‘footnotes’ [8] 10. If no information is available, clearly indicate that [6,17] 11. Rank evidence and recommendations [1,6,7] 12. ~~Don’t put implications in summary tables [8]; keep separate from review [6]~~ ^~~a~~^ 13. Noticeable hyperlinks to supporting documents (full review, data, individual studies, etc.) [1,2,5,5,7,11] 14. Clear referencing style to not confuse with numeric data[15] 15. Consistency between numbers in tables/text [12]^b^ 16. Avoid statistical information [5]^b^ 17. Succinct methods, data sources, review approach, and criteria [1]^b^   **Structure**   1. Concise [3–8] 2. Brief narrative report [9,10]* 3. Structured [1,4,7,9,10]* 4. Intuitive presentation [6]^a^ 5. High-level one page summary [5,11] 6. Consistent presentation [4] 7. Summary of findings (SoF) tables [5,6,8,10,12–14]*, allowing for qualitative data [8] in prominent positions [15]   **Typography**   1. Use bullet points [5,6,18] 2. Flag important information [6,8] by bolding/highlighting [6]^a^, don’t put in footnotes[8] 3. Greyscale-friendly colours[1]^b^ 4. Avoid dense information. Promote white space [1,5,9]   **Results, tables & figures**   1. Use variations in cell colour/fonts for multiple control group risks [15] 2. ~~Shade rows [1]~~^~~b~~^ 3. Decrease numeric/statistical data [4,5,15] 4. ~~Use absolute numbers, not probabilities [8]~~ 5. Present numbers in tables and/or visually [4]; use icons [5] or graphics [9]^b^[19] 6. Limit visual information to single table/image [4] 7. Balance visual and textual information [1]^b^ 8. ~~Use multiple columns [5]~~ 9. Don’t break tables over multiple pages [5,12,16] 10. ~~Avoid forest plots [5]~~ 11. Keep footnotes on same page as tables [8] |
| **Tailoring Information** |
| **Structure**   1. Standard formatting to aid familiarity with repeated exposure [7] 2. Flexibility in delivery (electronic/PDF, printable, not requiring internet) [7] 3. Easily extractable information to forward to colleagues and use personally [5,9] 4. ~~Structure question around condition first, then intervention [2]~~^~~a~~^ 5. ~~Present positive results first, then negative [8]~~^~~a~~^ 6. AMRaD format [6]^a^ 7. Avoid academic formatting [5] ^b^ 8. No more than 1 page [12]^c^ 9. No more than 3 pages [1]^b^ 10. Visual format might be more useful [2] ^e^   **Content**   1. Clarify audience [1]^b^ 2. Accommodate different learning styles [2] 3. Choice and control over the amount of detail received [2,5,7] 4. Consider interpretation aids for statistics [2]^a^ 5. Short summary with conclusions for key questions most helpful [18] ^a^ 6. ~~Use end user’s native language [9]~~^~~b~~^ 7. Title, key messages, link to more detail [9]^b^ 8. Don’t include methodology information [4]^f^ [5]^b^ 9. Provide inclusion and exclusion criteria [2]^d^ |
| **Contextualising Findings** |
| **Content**   1. Legal/political conditions in country/region [9]^b^ 2. Framed within local [2,7,12], national, [5,9] or broader context [12]^b^ 3. Implementation/application information [4–6] 4. ~~Cost analyses [7,12]~~ 5. Limitations of findings [13] 6. Ramifications of methodological approaches [13] 7. Recommendations for practice/policies [2,5,7,12,15] and future research needs [13] 8. Clinical scenario example [6,13] and bottom line [6]^a^ 9. Effective intervention details [5,15] to help implementation, (e.g., dosages [5,6], trade names [6]^a^, treatment duration/frequency [5,6], costs [6]^a^, settings [5,11], evaluators of treatments [6]^a^, prevalence estimates [5,8], population characteristics [2,5,6,11] 10. ~~Not interested in interventions with no effects or search results information [5]~~ ^~~b~~^ |
| **Quality of Evidence** |
| **Content**   1. Include quality assessment of evidence/study quality [1,2,4,13,16,20]* 2. Provide distinct explanations of rating scale (GRADE) [4,6,8,10,14,16,17,20]* 3. Detail how authors arrived at assessments of quality [9,10,20]* in footnotes [8] 4. Rank or group studies [1,6] 5. Appreciate methodology details and limitations [5]^c^ |
| **Trust in Producers and Summary** |
| **Content**   1. Include conflict of interest statements (of primary studies) [9]_b_ and summary producers [6] ^a^ 2. Include funding sources [5] 3. Include authors’ names [6,7,11,12] 4. Put logos on first page [1,7,9,12] 5. Include clear references [12]^b^ 6. Establish credibility of research evidence [7] |
| **Knowledge Required** |
| **Content**   1. Avoid field-specific or technical jargon (e.g., ‘scaling up’, ‘EBM’, ‘PICO’) [10–12] 2. Avoid abbreviations (e.g., RR for relative risk, CI for confidence intervals [15] 3. Provide information on nature of systematic review and standard steps [11–13,19]*   **Results, tables & figures**   1. ~~Define ‘no data available’ and reasons for empty cells [15]~~ 2. Define statistical terms [5,8,9,11,12,14,15]* 3. Define relative risk and confidence interval in forest plots [5] 4. ~~Forest plots are difficult to understand [3]~~ 5. Provide interpretation of statistical results [10,14,19]* 6. ~~Define column labels [8]~~ 7. ~~Avoid probabilities [4,8]~~ 8. Use similar table formats to aid readability with repeated exposure makes tables easier to read [15] |

**References**

1. Hartling L, Gates A, Pillay J, Nuspl M, Newton AS. Development and Usability Testing of EPC Evidence Review Dissemination Summaries for Health Systems Decisionmakers. Agency for Healthcare Research and Quality (US); 2018; Available from: http://ovidsp.ovid.com/ovidweb.cgi?T=JS&PAGE=reference&D=medp&NEWS=N&AN=30507111

2. Totten AM, Smith C, Dunham K, Jungbauer RM, Graham E. Improving Access to and Usability of Systematic Review Data for Health Systems Guidelines Development. Agency for Healthcare Research and Quality (US); 2019; Available from: http://ovidsp.ovid.com/ovidweb.cgi?T=JS&PAGE=reference&D=medp&NEWS=N&AN=31013017

3. Babatunde OO, Tan V, Jordan JL, Dziedzic K, Chew-Graham CA, Jinks C, et al. Evidence flowers: An innovative, visual method of presenting “best evidence” summaries to health professional and lay audiences. Research Synthesis Methods. 2018;9:273–84.

4. Buljan I, Tokalić R, Roguljić M, Zakarija-Grković I, Vrdoljak D, Milić P, et al. Comparison of blogshots with plain language summaries of Cochrane systematic reviews: a qualitative study and randomized trial. Trials. 2020;21:426.

5. Marquez CJ Alekhya Mascarenhas; Jassemi, Sabrina; Park, Jamie; Moore, Julia E; Blaine, Caroline; Bourdon, Gertrude; Chignell, Mark; Ellen, Moriah E; Fortin, Jacques; Graham, Ian D; Hayes, Anne; Hamid, Jemila S; Hemmelgarn, Brenda R; Hillmer, Michael P; Holmes, Bev; Holroyd-Leduc, Jayna; Hubert, Linda; Hutton, Brian; Kastner, Monika; Lavis, John N; Michell, Karen; Moher, David; Ouimet, Mathieu; Perrier, Laure; Proctor, Andrea; Noseworthy, Tom; Schuckel, Victoria; Stayberg, Sharlene; Tonelli, Marcello; Tricco, Andrea C; Straus, Sharon E. Enhancing the uptake of systematic reviews of effects: what is the best format for health care managers and policy-makers? A mixed-methods study. 2018;13:84-NA.

6. Perrier LK M Ryan; Straus, Sharon E. An iterative evaluation of two shortened systematic review formats for clinicians: a focus group study. 2014;21:e341-6.

7. Dobbins MT Helen; O’Brien, Mary Ann; Duggan, Melissa. Use of systematic reviews in the development of new provincial public health policies in Ontario. 2004;20:399–404.

8. Mustafa R, Wiercioch W, Brozek J, Lelgemann M, Buehler D, Garg A, et al. Enhancing the acceptance and implementation of grade summary tables for evidence about diagnostic tests. BMJ Quality and Safety. 2013;22:A36.

9. Busert LK, Mütsch M, Kien C, Flatz A, Griebler U, Wildner M, et al. Facilitating evidence uptake: Development and user testing of a systematic review summary format to inform public health decision-making in German-speaking countries. Health Research Policy and Systems [Internet]. 2018;16. Available from: https://www.scopus.com/inward/record.uri?eid=2-s2.0-85049782278&doi=10.1186%2fs12961-018-0307-z&partnerID=40&md5=8a60b2081f09fd2655dac0ddecb23467

10. Opiyo N, Shepperd S, Musila N, Allen E, Nyamai R, Fretheim A, et al. Comparison of Alternative Evidence Summary and Presentation Formats in Clinical Guideline Development: A Mixed-Method Study. PLoS ONE [Internet]. 2013;8. Available from: ://WOS:000315210400056

11. Steele R. Mental health clinicians views of summary and systematic review utility in evidence-based practice. Health Information and Libraries Journal [Internet]. Available from: ://WOS:000627057300001

12. Rosenbaum SE, Glenton C, Wiysonge CS, Abalos E, Mignini L, Young T, et al. Evidence summaries tailored to health policy-makers in low- and middle-income countries. Bull World Health Organ. 2011;89:54–61.

13. Hartling L, Guise JM, Hempel S, Featherstone R, Mitchell MD, Motu’apuaka ML, et al. Fit for purpose: Perspectives on rapid reviews from end-user interviews. Systematic Reviews [Internet]. 2017;6. Available from: https://www.scopus.com/inward/record.uri?eid=2-s2.0-85013130994&doi=10.1186%2fs13643-017-0425-7&partnerID=40&md5=d0ea651ce13e9b75e702f2b5a9e822cc

14. Rosenbaum SG Claire; Oxman, Andrew D. Summary-of-findings tables in Cochrane reviews improved understanding and rapid retrieval of key information. 2010;63:620–6.

15. Rosenbaum SG Claire; Nylund, Hilde Kari; Oxman, Andrew D. User testing and stakeholder feedback contributed to the development of understandable and useful Summary of Findings tables for Cochrane reviews. 2010;63:607–19.

16. Yepes-Nunez JJ, Li SA, Guyatt G, Jack SM, Brozek JL, Beyene J, et al. Development of the summary of findings table for network meta-analysis. Journal of Clinical Epidemiology. 2019;115:1–13.

17. Perrier LK M Ryan; Straus, Sharon E. A usability study of two formats of a shortened systematic review for clinicians. 2014;4:e005919-NA.

18. Newberry SJ, Shekelle PG, Vaiana M, Motala A. Reporting the Findings of Updated Systematic Reviews of Comparative Effectiveness: How Do Users Want To View New Information? Agency for Healthcare Research and Quality (US); 2013; Available from: http://ovidsp.ovid.com/ovidweb.cgi?T=JS&PAGE=reference&D=medp&NEWS=N&AN=23785728

19. Buljan I, Malički M, Wager E, Puljak L, Hren D, Kellie F, et al. No difference in knowledge obtained from infographic or plain language summary of a Cochrane systematic review: three randomized controlled trials. J Clin Epidemiol. 2018;97:86–94.

20. Carrasco-Labra A, Brignardello-Petersen R, Santesso N, Neumann I, Mustafa RA, Mbuagbaw L, et al. Improving GRADE evidence tables part 1: a randomized trial shows improved understanding of content in summary of findings tables with a new format. Journal of Clinical Epidemiology. Elsevier; 2016;74:7–18.
