# Supplementary material for: Co-design workshops to develop evidence synthesis summary formats for use by clinical guideline development groups
Source: Syst Rev. 2024 Mar 27;13:97. doi: 10.1186/s13643-024-02518-z (PMC10967093; doi:10.1186/s13643-024-02518-z)
Supplement: Supplementary file 3 — Additional file 3. Topic Guide. [file 13643_2024_2518_MOESM3_ESM.docx]

Topic Guide

Contents

[Topic Guide 1](#_Toc137035668)

[AGENDA AND LOGISTICS 2](#_Toc137035669)

[EXAMPLES OF SUMMARIES 2](#_Toc137035670)

[AGENDA AND LOGISTICS 3](#_Toc137035671)

[PROJECT BACKGROUND 4](#_Toc137035672)

[GROUND RULES AND TECH CHECK 5](#_Toc137035673)

[RECOMMENDATIONS WITH MIXED METHODS SUPPORT – QUALITY OF EVIDENCE AND KNOWLEDGE REQUIRED 6](#_Toc137035674)

[RECOMMENDATIONS WITH MIXED METHODS SUPPORT – PRESENTING INFORMATION 7](#_Toc137035675)

[RECOMMENDATIONS WITH STRONG SUPPORT -- PRESENTING INFORMATION 7](#_Toc137035676)

[RECOMMENDATIONS WITH STRONG SUPPORT -- CONTEXTUALISING AND TAILORING INFORMATION 8](#_Toc137035677)

[RECOMMENDATIONS WITH STRONG SUPPORT -- TRUST IN PRODUCER AND SUMMARY 9](#_Toc137035678)

[EXAMPLE FORMATS 10](#_Toc137035679)

[VOTING SLIDE (SLIDE 16) 11](#_Toc137035680)

[ESSENTIAL INFORMATION FOR 1, 3, AND 5 PAGE SUMMARIES 11](#_Toc137035681)

[WRAP UP 12](#_Toc137035682)

| **SLIDE** | **Text** | **Additional prompts or text for chat** |
| --- | --- | --- |
| AGENDA AND LOGISTICS (SLIDE 1)  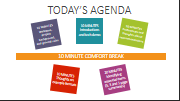 | Hi everyone, we’re going to get started. [FACILITATOR RECORDS NOW.] I’m X, I’ll be chairing today’s session. [ONE SENTENCE INTRO ON WHO CHAIR IS] I’m being joined by Y AND Z who will be acting as our moderators and tech support. [ONE SENTENCE INTRO FROM MODERATOR AND TECH SUPPORT]  So we have around 90 minutes today to chat -- which I’m sure once we get into it will just fly by. Hopefully everyone can see today’s agenda on the screen. We’ll be using some interactive tools today to help the discussion so if you have two screens, we’d recommend using both. If you are having technical difficulties, pop a message in the chat and MODERATOR *(or RA if available)* can try to assist.  So, we’re going to start off with a brief summary about the work we’ve been doing recently which we’re going to hopefully build upon with you. You’ve all been engaged with this project because you’ve been involved in some sort of expert advisory group, the clinical guideline development process, or have relevant expertise.  The goal for today is for everyone to share their thoughts and experiences with evidence synthesis summary formats and how they can be improved. Hopefully with your suggestions and help, we can design a few prototype summary formats to test and eventually integrate into regular use. | COPY-PASTE INTO CHAT WAITING ROOM: Hello all, we’ll get started shortly. As a reminder, if we have not received your informed consent form yet, we cannot let you join. Please email [[x]](mailto:rmurray@hiqa.ie) your copy if you have not already done so. Additionally, if you have not completed your About You form, please do so now here: *LINK*  *or*  Hello all, we’ll get started shortly. As a reminder, if you have not completed your About You form, please do so now here: *LINK* |
| EXAMPLES OF SUMMARIES (SLIDE 2a or 2b)  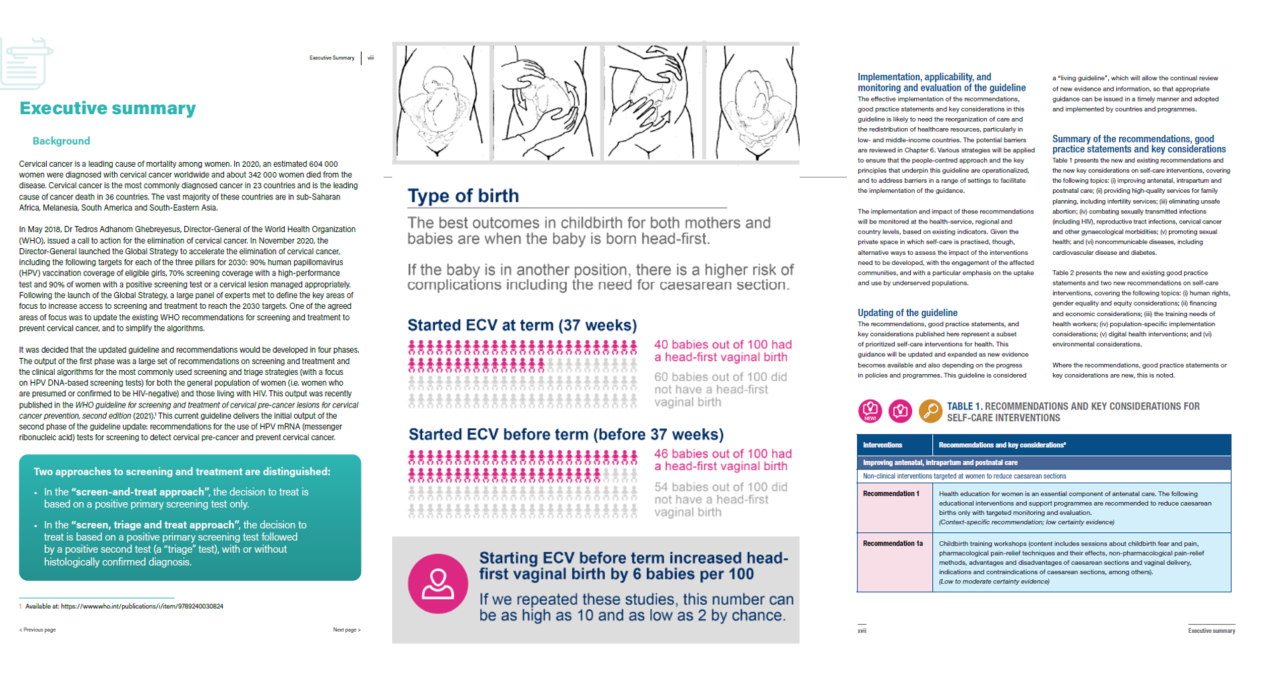  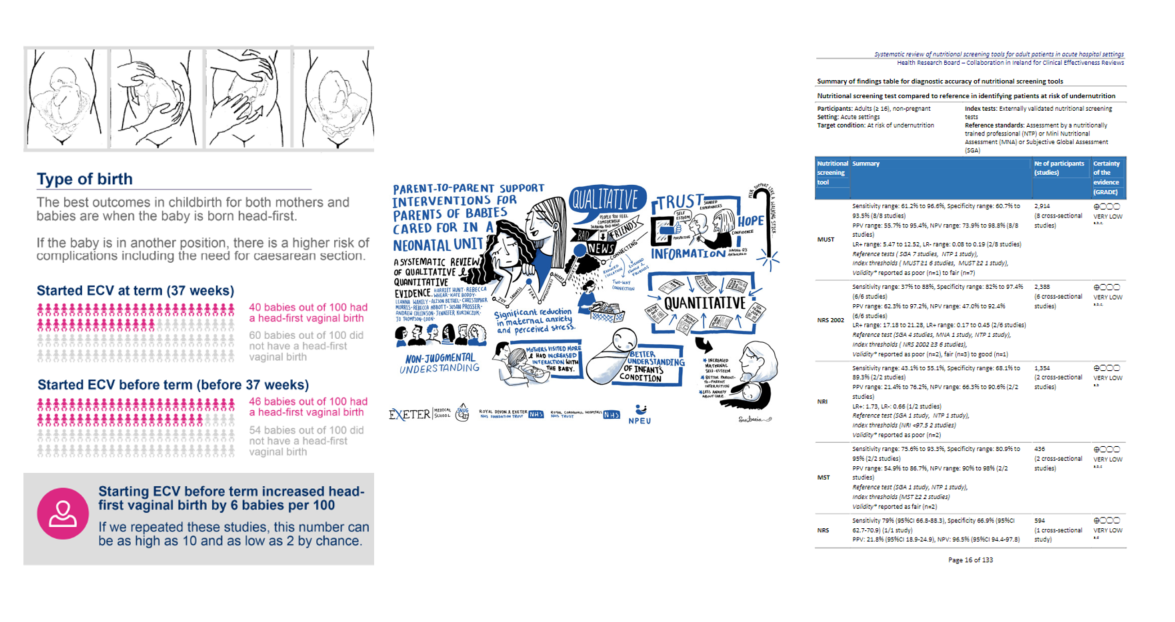 | Some examples of summary formats that are currently being used were presented to you on the ‘About You’ background form which was asked you to complete. So a quick reminder to complete that if you haven’t done so already (the link will be in the chat if you need it). But as a refresher, some examples are 1, 3 or 5 page summaries, abstracts, summary of findings tables, policy briefs, infographics, podcasts, visual summaries, interactive dashboards and the list goes on…  On the screen are just three examples of executive summaries which we’ll revisit later. As you can see they take different approaches with formatting and with using visuals or infographics. | Microsoft Forms: ‘About You’ *LINK* |
| AGENDA AND LOGISTICS (SLIDE 3)  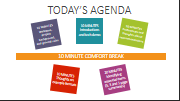 | As many of you likely know from being engaged with synthesizing the evidence yourself, making summary formats, or being involved in a group that’s talking about and trying to make decisions based on these summaries, things can get quite complex and nuanced, so we’re splitting up the conversation into different topics areas. We’ll start by sharing some of the main findings from our review and then get your thoughts on these different recommendations for what to include in a summary format (keeping in mind this may differ from person to person). We’ll then have a short comfort break and finish off the day honing in on the essentials to include in 1, 3 and 5 page summaries. |  |
| PROJECT BACKGROUND (SLIDE 4)  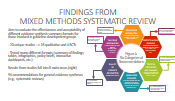 | First off, to bring you up to speed, we’ll give you a bit of context to how we got here. Most recently, we conducted a mixed methods systematic review which explored what was out there in terms of different types of evidence synthesis summary formats and how those involved in clinical guideline development (like yourselves) viewed these different formats.  Being a mixed methods review, we searched for information from quantitative studies like randomised controlled trials and qualitative studies like interviews and focus groups. We found 20 different studies, which looked at a variety of different formats like summary of findings tables, policy briefs, infographics, interactive dashboards, etc. We pooled together all of the results from these studies and created 6 main areas that the findings fell into. These included things like comments on the content and structure of information -- to things like what kind of knowledge base was required to actually use the information that was presented.  There was no clear summary format that was preferred by the participants in the studies but for practicality’s sake today we’re going to focus mostly on the ‘written’ formats like tables, and figures, 1, 3, or 5 page summaries. Keeping in mind the whole that there will always be a full technical report available. We’re just focusing on what should go in the summary.  So our starting point today is going to be linked to these 94 recommendations which fell into these 6 different categories. We’re hoping to focus on the recommendations that had a stronger evidence base to support them and also those which maybe didn’t have as strong of a consensus so we can chat about and explore them more. So that’s the basis of what we’ve been doing and the plan for today… if anyone has any questions before we jump into the tech and introductions?  **PAUSE FOR QUESTIONS** |  |
| GROUND RULES AND TECH CHECK (SLIDE 5 AND 6)  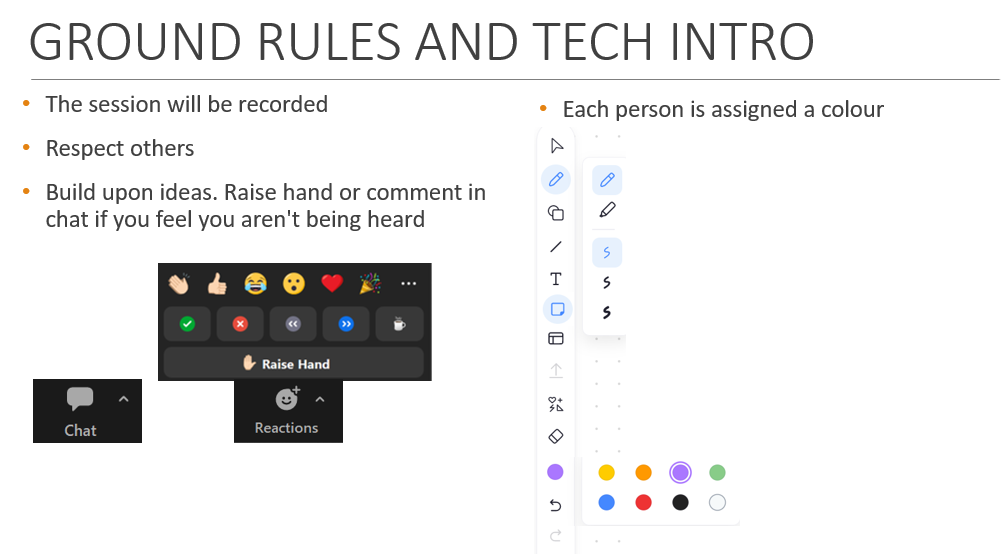  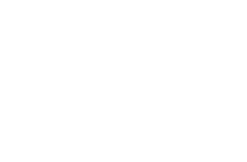 | Okay, some brief ground rules so things go as smoothly as possible...  First off, we’re recording today’s session. If there’s feedback, please mute yourself and if the signal is bad, please feel free to turn off your camera. This is a group where people may have shared experiences or very different opinions so please try to respect each other’s perspectives and ideas and try to avoid interrupting and talking over people if possible.  Building upon each other’s ideas is great and there’s no such thing as a stupid question so please if you’re ever feeling lost or not heard, you can send a message in the chat or you can use the raise hand function (under reactions at the bottom of your screen) to make sure we see that you would like to talk. Discussions may meander from time to time so we’ll try to take your feedback into account but we might try to redirect the conversation back to the topic at hand if we ever get off track.  Now to start, we’re going to do a quick activity so you can both practice the drawing, stamp, and text functionalities on here and so we can all get to know each other. You were all assigned a colour and shape to use in the final reminder email that we sent. In case you forgot, they will be put in the chat. So for the entire discussion today, please use your colour and shape where applicable so we can tell who is saying what.  On your screen there should be an annotation toolbar which will allow you to directly add content to the slides. As I’m the host, my toolbar might look a bit different than yours but essentially if you want to add text to the slide, you can select the text. And if you want to add a shape to the slide you can find your shape under stamp or under draw.  **COLOUR AND SHAPE ASSIGNMENT AND INTRODUCTION ACTIVITY**  For a little practice, you can start by typing your name anywhere on the screen in the colour assigned to you. And then add your stamp next to your name. As people are done, we’ll start with introductions to each other. If you can share a few sentences about your background or experience with guideline development, evidence synthesis, or developing summary documents. | NAME – **STAR**  NAME **- HEART**  NAME – **CHECK MARK**  NAME - **X**  NAME – **QUESTION MARK**  NAME –  **FILLED CIRCLE** |
| RECOMMENDATIONS WITH MIXED METHODS SUPPORT – QUALITY OF EVIDENCE AND KNOWLEDGE REQUIRED (SLIDE 7)  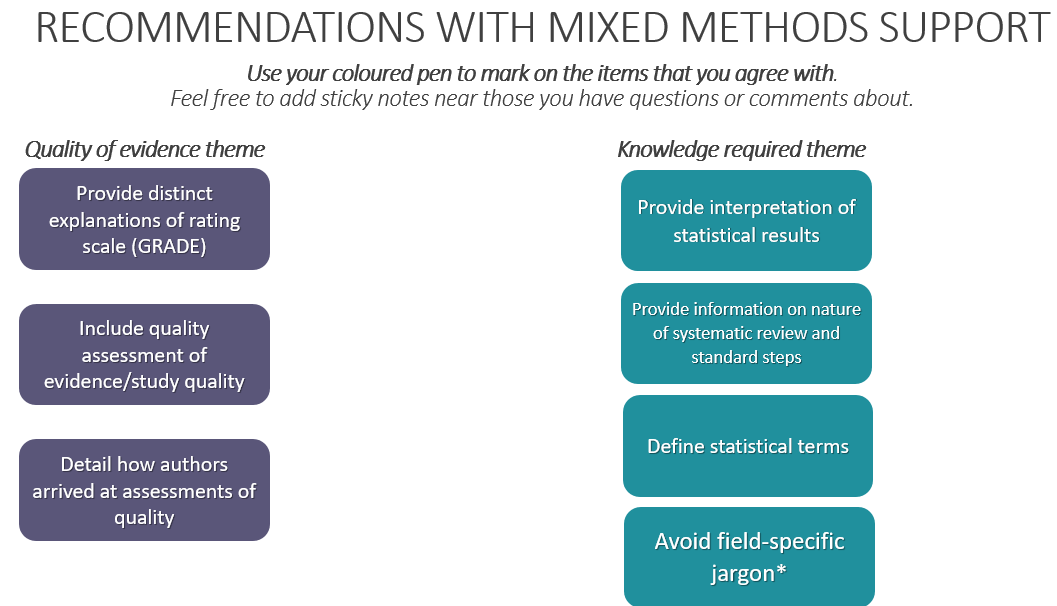 | Now that we all know who’s who and how to add shapes and text, we’re going to start off with a few of those recommendations from our review that we found the most support for. These recommendations were supported by both quantitative and qualitative evidence, and largely dealt with, the quality of evidence, the knowledge required to use a summary, and how to present the information.  **ACTIVITY:**  ***Please use your shape to mark the items that you agree with, that is, that you feel this item would be essential in a summary of any evidence synthesis. Feel free to add text near those you have questions or comments about.***  Does anyone want to kick us off with some thoughts on any of these? *(Start with ones with least consensus. If no speakers, focus on any outliers within each category.)*   - Did anyone have any initial comments about the three items in purple about quality of evidence? - Did anyone have any initial comments about the items in teal about knowledge?   Any last comments on anything related to these two themes before we move on? | *Within the quality of evidence theme…*   - How important do you think it is to provide a reader with ranked evidence and recommendations? (prioritisation exercise) - How much information do you want about how the authors arrived at their assessments? Do you think this information should go in the footnotes or somewhere else? (prioritisation exercise)   Within the knowledge required theme…   - When defining statistical terms, how much information do you want or need? Would an interpretation aid for statistics be helpful? (prioritisation exercise) Where do you think is the best place for this? - Are there certain terms that you think are especially important to define? (e.g., relative risk, confidence intervals, any info around forest plots?) (prioritisation exercise) - How do you feel about statistical abbreviations? (prioritisation exercise) |
| RECOMMENDATIONS WITH MIXED METHODS SUPPORT – PRESENTING INFORMATION (SLIDE 8)  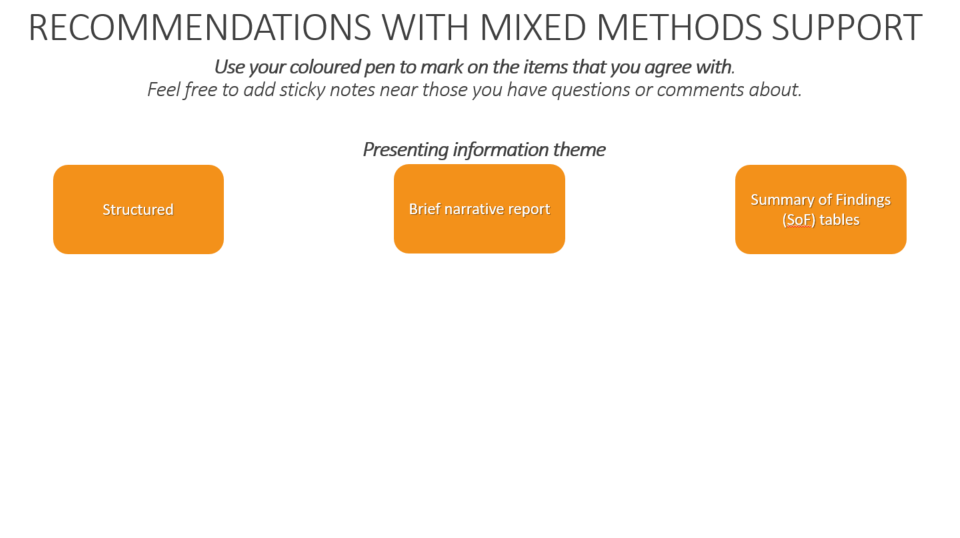 | Now these have to deal with how the information is presented on the page.  **ACTIVITY:**  ***Please use your shape to mark the items that you agree with, that is, that you feel this item would be essential in a summary of any evidence synthesis. Feel free to add text near those you have questions or comments about.***   - Did anyone have any initial comments these three items? For example, ‘structured’ could be viewed as a vague term so when you think of a structured summary, what does that look like visually to you? - What kind of information would you want included in a narrative summary?   Any last comments before we move on?  ***.*** | *Within the presenting information theme…*   - Can you suggest ways to emphasize the information that is important to you in a summary? (literature review) - One can provide some structure by using prominent subheadings -- what would you like those to be? (prioritisation exercise) - How would you feel about a typical academic format like IMRaD being used? (introduction, methods, results, and discussion) - How important do you think it is to report what worked and what didn’t? (prioritisation exercise) |
| **BASED ON TIMING,**  **CHOOSE EITHER ONLY SLIDE 9**  **OR**  **SLIDES 10 AND 11** | | |
| RECOMMENDATIONS WITH STRONG SUPPORT -- PRESENTING INFORMATION (SLIDE 9)  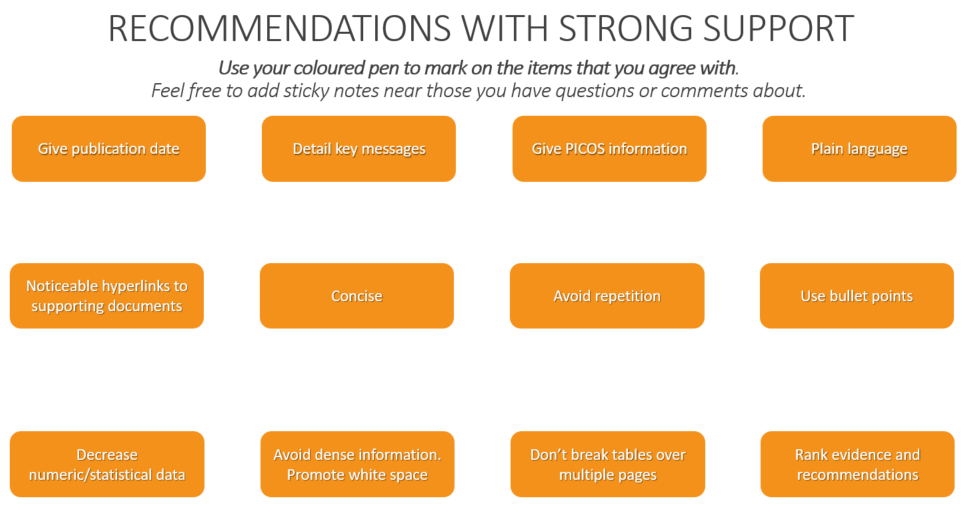 | Here are some examples of items about presenting information that had strong support from at least 3 studies but not support from both the trials and qualitative evidence. Some of these may have been discussed previously and some may have not been mentioned.  **ACTIVITY:**  ***Please use your shape to mark the items that you agree with, that is, that you feel this item would be essential in a summary of any evidence synthesis. Feel free to add text near those you have questions or comments about.***  Any last comments before we move on? | - How useful is a summary that presents the results in bullets, tables, and figures? - Say you’re involved in multiple syntheses or groups, how important is it to you that the presentation of information is consistent? So with repeated exposure, the format is the same? (prioritisation exercise) |
| RECOMMENDATIONS WITH STRONG SUPPORT -- CONTEXTUALISING AND TAILORING INFORMATION (SLIDE 10)  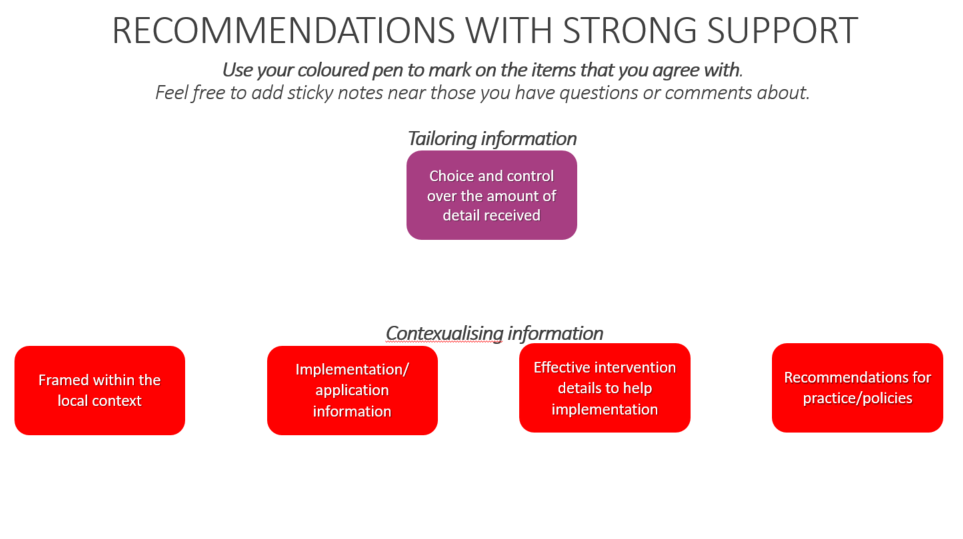 | In the interest of time, there are other items with strong support from the presenting information theme but we’re gonna switch gears and talk about tailoring information and providing context to the results. So, here are a few recommendations that have been previously proposed.   - For example, how important do you think it is to give recommendations for clinical care, policies, or implementation within a summary? - And how much control do you want over the level of detail that you’re getting within a summary?   **ACTIVITY:**  ***Please use your shape to mark the items that you agree with, that is, that you feel this item would be essential in a summary of any evidence synthesis. Feel free to add text near those you have questions or comments about.***  Any last comments before we move on? | - Do you think that an example clinical scenario should be presented? (prioritisation exercise) - What would you be willing to trade-off to get your summary in a timely fashion? (mixed methods systematic review) - How important do you think it is that limitations of findings be included on a 1 page summary? A longer format? (prioritisation exercise) - In terms of ramifications of methodological approaches (e.g., a rapid review or different types of syntheses done) is this something that you would like included? (prioritisation exercise) - How important is it to you that the format is flexible (e.g., doesn’t require internet, printable, PDF)? What format would you prefer the summary to be in? (prioritisation exercise) |
| RECOMMENDATIONS WITH STRONG SUPPORT -- TRUST IN PRODUCER AND SUMMARY (SLIDE 11)  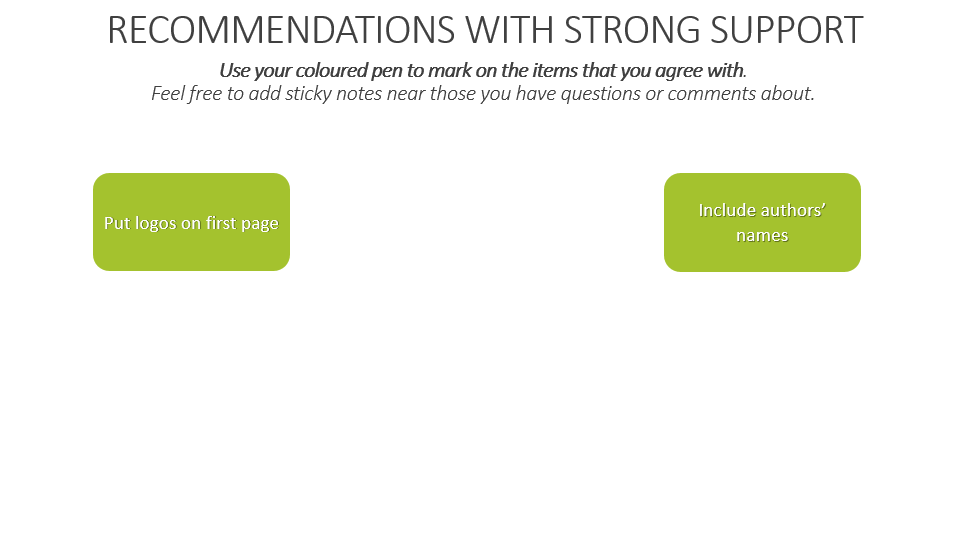 | Lastly, some recommendations pertained to trusting the producers of the summary or the summary itself. A couple recommendations are presented here**…**  **ACTIVITY:**  ***Please use your shape to mark the items that you agree with, that is, that you feel this item would be essential in a summary of any evidence synthesis. Feel free to add text near those you have questions or comments about.***  Any last comments before we move on? | - What pieces of information would you look for to tell us whether you would use it and/or trust it? (logos? All author names? Funding sources? Just the org name?) - Do you want information about the conflicts of interest of the original studies? The summary producers? (prioritisation exercise) - How important is your relationship with the producer of the evidence synthesis product? (in terms of credibility) |
| **COMFORT BREAK (SLIDE 12)** | | |
| EXAMPLE FORMATS (SLIDES 13a, 14a, 15a or 13b, 14b, 15b)  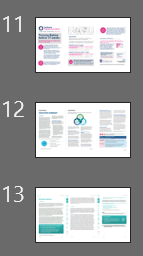  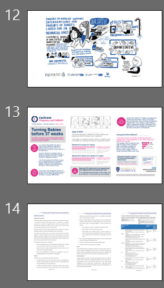 | Welcome back, I hope everyone had some time to stretch their legs and get a cup of tea or some water. Now moving forward from all our earlier discussions, we’re going to spend the rest of the time building the pieces of a format that we ideally would like to have one day. To kick off, we will revisit the few example summaries that you saw earlier which are from different organisations. This will hopefully give you a sense of some different options out there currently.  *The first format is from Cochrane and it focuses on the effects of trying to turn unborn babies before the end of pregnancy. This format is not really amenable to printing, it is one long pdf which you can scroll through. A few screenshots of the ‘pages’ are shown here. It has visual representations of the data, some images, and logos of the creators at the end. (13a and 14b)*  *Next, we have an executive summary from the World Health Organisation on self-care interventions. This summary is actually of the guideline itself so keep that in mind. It is around 6 pages followed by recommendations in a table with italicised notes at the end indicating the quality of evidence to support them. It contains a few graphics, subheadings, and bullet points. (14a)*  *And lastly, a guideline on screening for cervical cancer. This summary has more text than the others, some of which is hyperlinked to allow one to easily hop around different part of the document, and it has key messages and recommendations separated out in the coloured boxes. (15a)*  *First off, this is a very visual representation of a systematic review of the quantitative evidence. This one is created by a design agency. There's a few things going on here with the full list of authors, university logos and perhaps funders.(13b)*  *Next, we have an executive summary from the HRB CICER group. This one is on nutritional screening tools for adults in acute hospital settings. This executive summary is 9 pages and provides some subheadings, covers multiple review questions, and has a few summary of findings tables in it. (15b)* | - What is your overall impression of these formats? Are there certain characteristics that really stand out to you? Is there anything about the formats you like (don’t like)? Why? - What are three things you like best about the document? - What are the three things you like least about the document? - Order of operations? What do you read first? What catches your attention? - Would you make any changes to the appearance of x? (If yes, what would you change?) - Which improvements would you suggest? - What is the reason for your preference? |
| VOTING SLIDE(SLIDE 16) 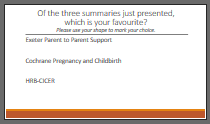 | **POLL ACTIVITY**  **PARTICIPANTS CHOOSE FAVORITE FORMAT FROM 3 PRESENTED OPTIONS AND DISCUSS ASPECTS THEY LIKED AND DIDN’T LIKE (PROMPTS ABOVE)** |  |
| ESSENTIAL INFORMATION FOR 1, 3, AND 5 PAGE SUMMARIES (SLIDE 17 and 18 )  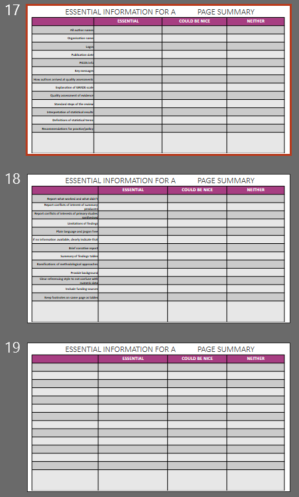 | Now putting everything together from earlier, what we liked, what we didn’t like, some visual examples of how others have done things, we’re going to try adding the items that you would like to see in an ideal summary document. Keep in mind that there will always be a full technical report but we are focused on what information you would like to see included in a condensed summary of that full report. In this case, what is essential to include in a x page summary.  **ACTIVITY: There are items on the screen which we may have previously discussed. As you’ll see the list is the same in each column so you can only agree with the 1 placement. For example, if you think it is essential that logos should be on a 1 page summary, put your stamp there. If you think a full list of authors could be not but perhaps not essential, you would put your stamp in that column.**  Building upon that first page, we’ll take forward the essential items obviously but we’ll explore what you think should be included when you have a bit more space. In this case, EITHER 3 OR 5 PAGES.  So what items would you like to see in a longer 3 or 5 page summary document? |  |
| **Based on time, do second slide (has different options) or give them extra space (i.e., 1 🡪 3 pages, 3 pages 🡪 5)** | | |
| WRAP UP | Do you have any other feedback, comments, or concerns regarding any of the materials we have discussed today?  Thanks to everyone for participating today. You all have our contact details so please feel free to get in touch if there’s any additional information you’d like to share with us or if you have any questions. We are doing a few of these groups and then combining all of the feedback to make some draft summary formats. At that time, we may be in touch with you again to see if you’d like to share your thoughts what we design. But in the meantime I hope you all have a lovely rest of your day/evening/week. Thanks again (insert awkward Zoom hand wave here.) Fin. |  |
