# Supplementary material for: Co-design workshops to develop evidence synthesis summary formats for use by clinical guideline development groups
Source: Syst Rev. 2024 Mar 27;13:97. doi: 10.1186/s13643-024-02518-z (PMC10967093; doi:10.1186/s13643-024-02518-z)
Supplement: Supplementary file 4 — Additional file 4. Workshop Slideset. [file 13643_2024_2518_MOESM4_ESM.pptx]

## Slide 1
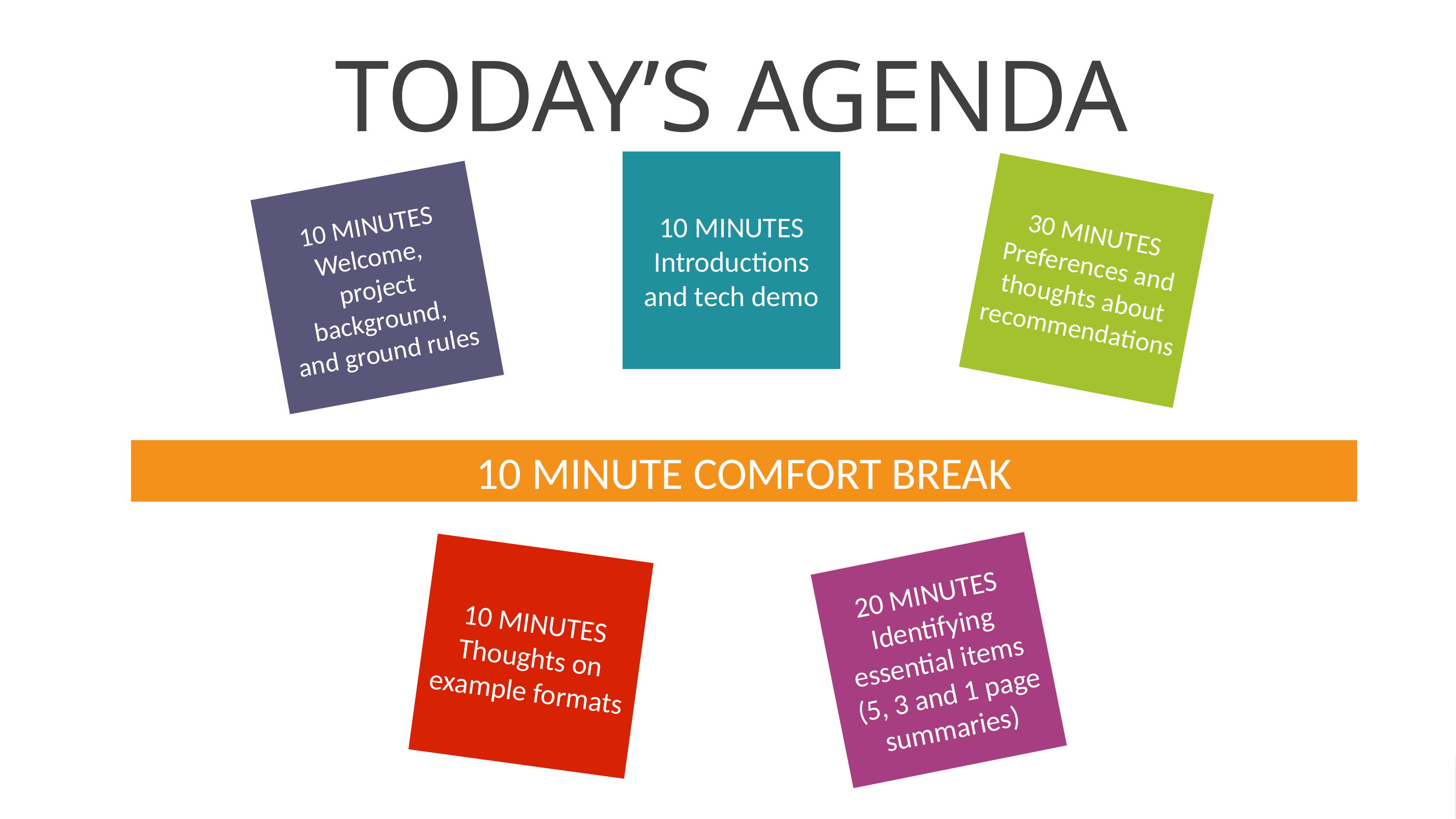

# TODAY’S AGENDA
10 MINUTES
Introductions and tech demo
30 MINUTES
Preferences and thoughts about recommendations
10 MINUTES
Welcome,
project background,
and ground rules
10 MINUTE COMFORT BREAK
10 MINUTES
Thoughts on example formats
20 MINUTES
Identifying essential items
 (5, 3 and 1 page summaries)

## Slide 2
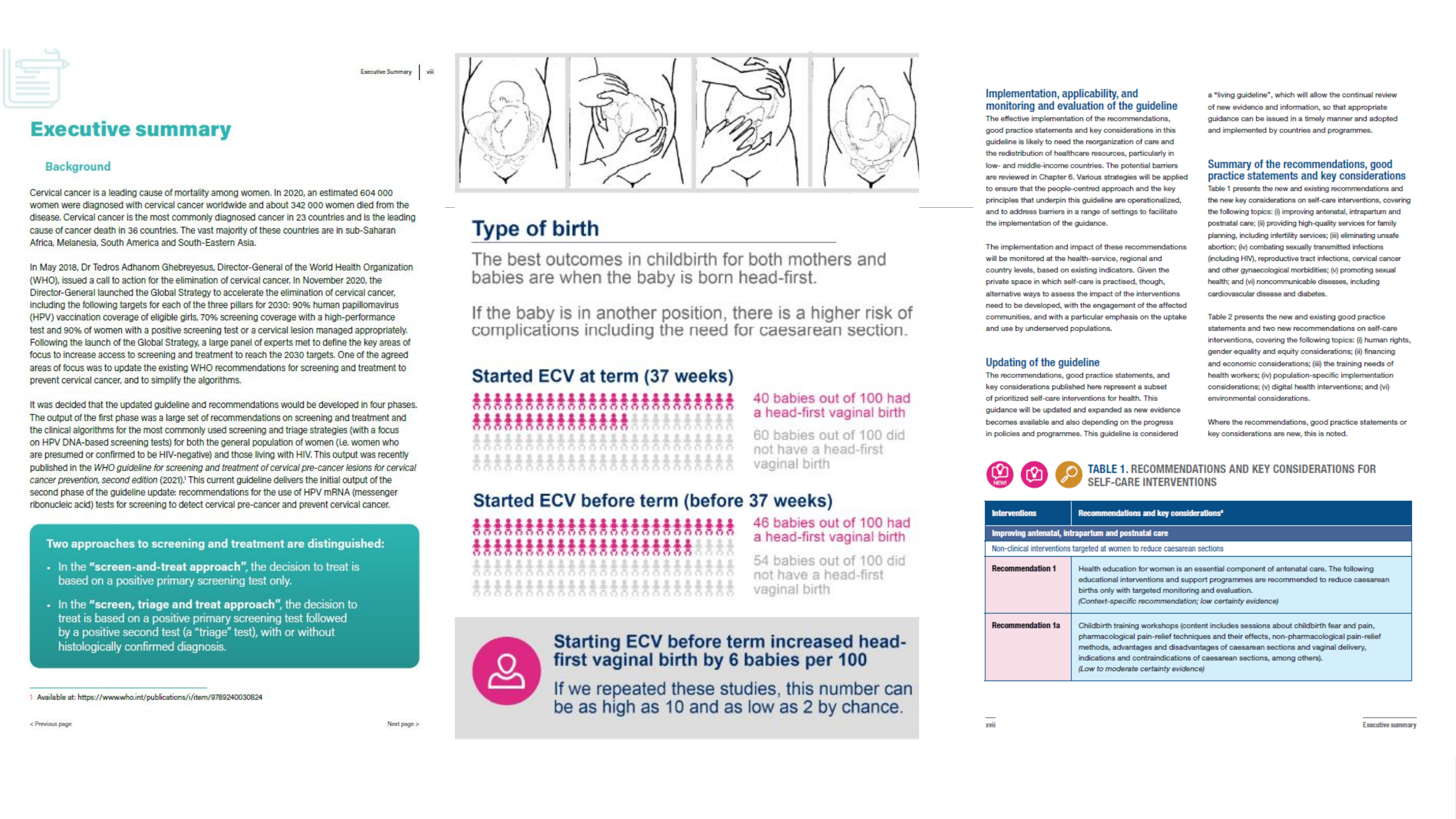

## Slide 3
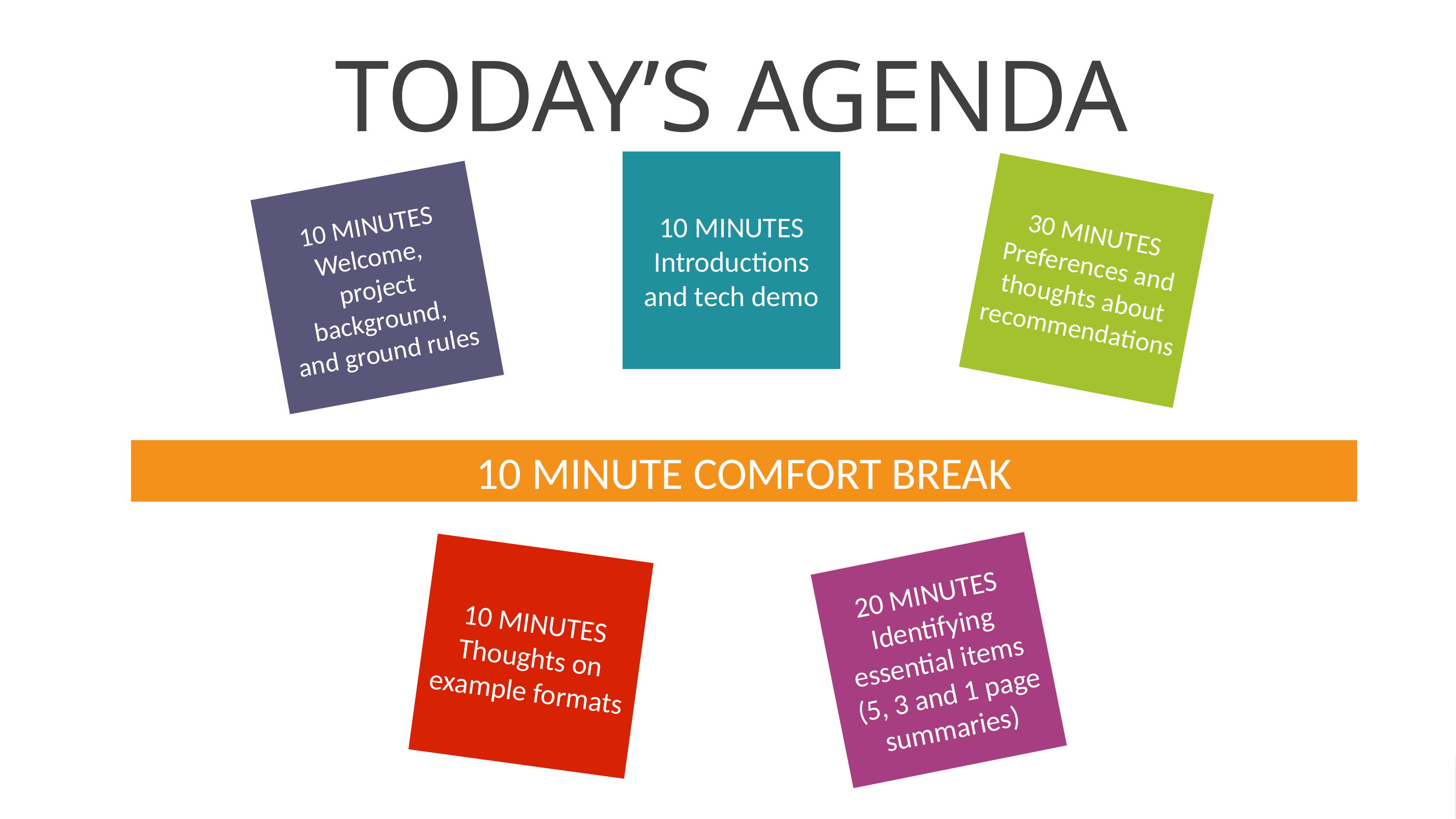

# TODAY’S AGENDA
10 MINUTES
Introductions and tech demo
30 MINUTES
Preferences and thoughts about recommendations
10 MINUTES
Welcome,
project background,
and ground rules
10 MINUTE COMFORT BREAK
10 MINUTES
Thoughts on example formats
20 MINUTES
Identifying essential items
 (5, 3 and 1 page summaries)

## Slide 4
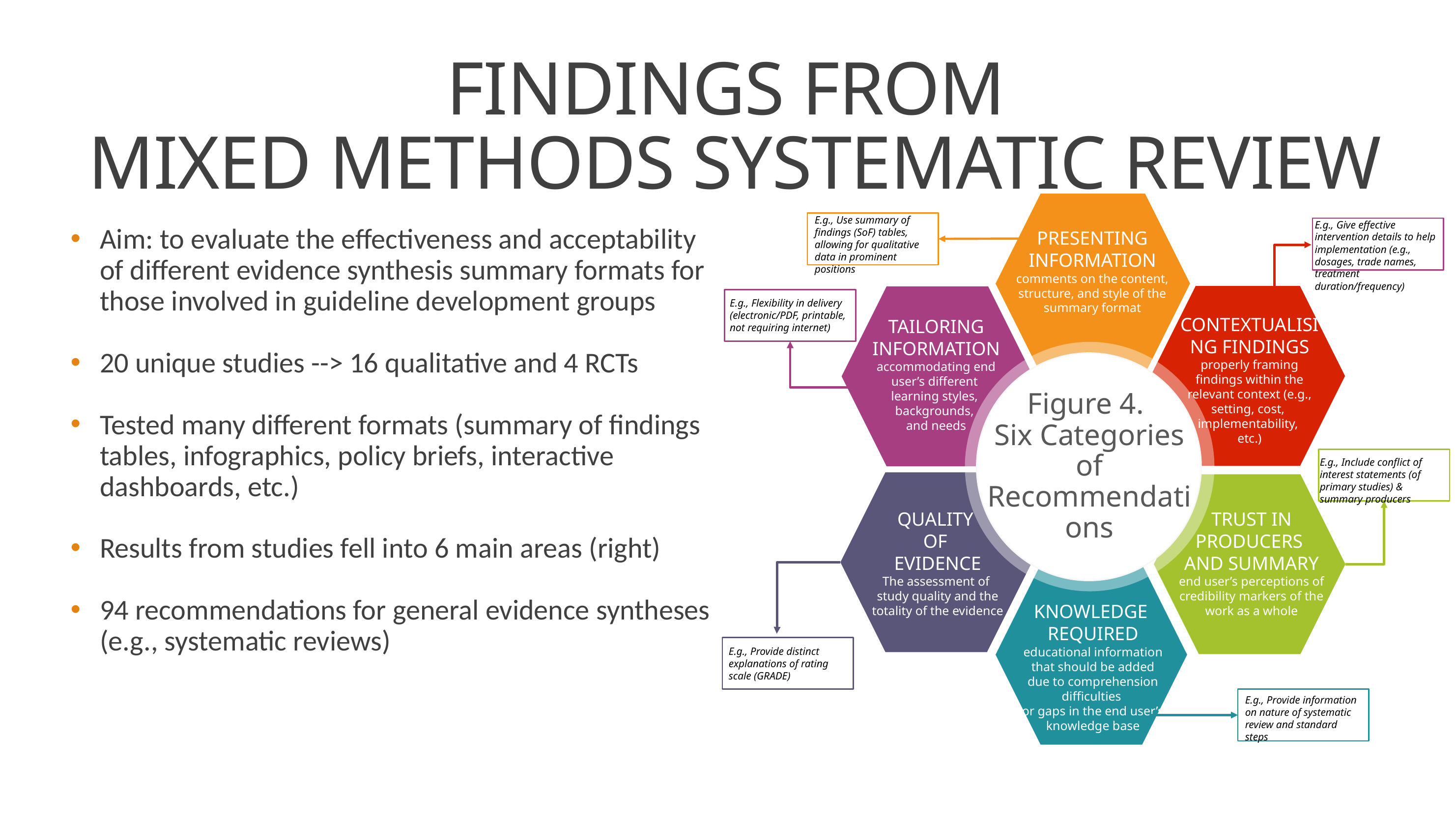

# FINDINGS FROM MIXED METHODS SYSTEMATIC REVIEW
E.g., Use summary of findings (SoF) tables, allowing for qualitative data in prominent positions
E.g., Give effective intervention details to help implementation (e.g., dosages, trade names, treatment duration/frequency)
Aim: to evaluate the effectiveness and acceptability of different evidence synthesis summary formats for those involved in guideline development groups
20 unique studies --> 16 qualitative and 4 RCTs
Tested many different formats (summary of findings tables, infographics, policy briefs, interactive dashboards, etc.)
Results from studies fell into 6 main areas (right)
94 recommendations for general evidence syntheses (e.g., systematic reviews)
PRESENTING INFORMATION
comments on the content, structure, and style of the summary format
E.g., Flexibility in delivery (electronic/PDF, printable, not requiring internet)
CONTEXTUALISING FINDINGS
properly framing findings within the relevant context (e.g., setting, cost,
implementability,
etc.)
TAILORING INFORMATION
accommodating end user’s different
learning styles,
backgrounds,
and needs
Figure 4.
Six Categories of Recommendations
E.g., Include conflict of interest statements (of primary studies) & summary producers
QUALITY
OF
EVIDENCE
The assessment of
study quality and the totality of the evidence
TRUST IN PRODUCERS
AND SUMMARY
end user’s perceptions of credibility markers of the work as a whole
KNOWLEDGE
REQUIRED
educational information that should be added due to comprehension difficulties
or gaps in the end user’s
knowledge base
E.g., Provide distinct explanations of rating scale (GRADE)
E.g., Provide information on nature of systematic review and standard steps

## Slide 5
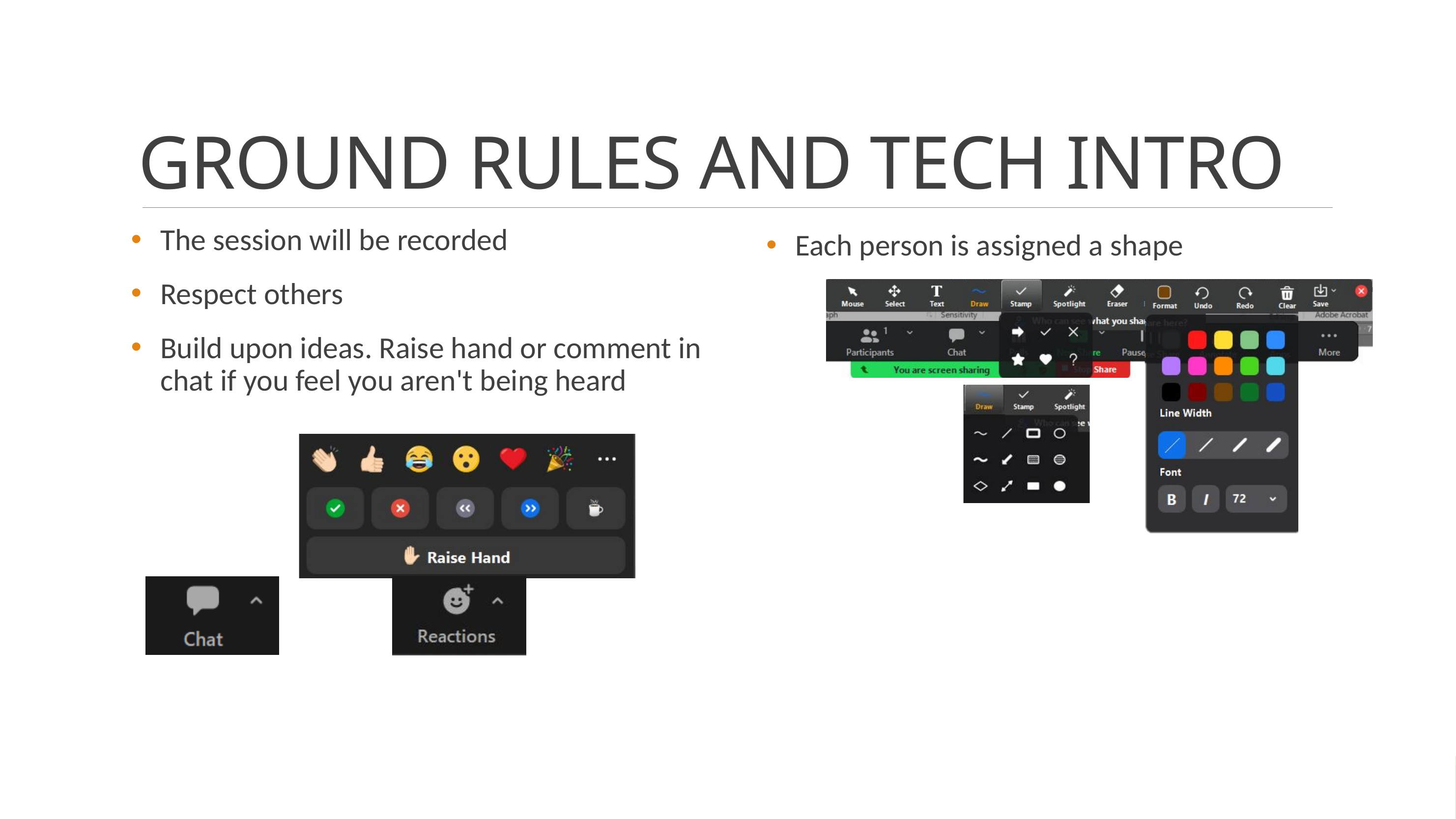

# GROUND RULES AND TECH INTRO
The session will be recorded
Respect others
Build upon ideas. Raise hand or comment in chat if you feel you aren't being heard
Each person is assigned a shape

## Slide 6
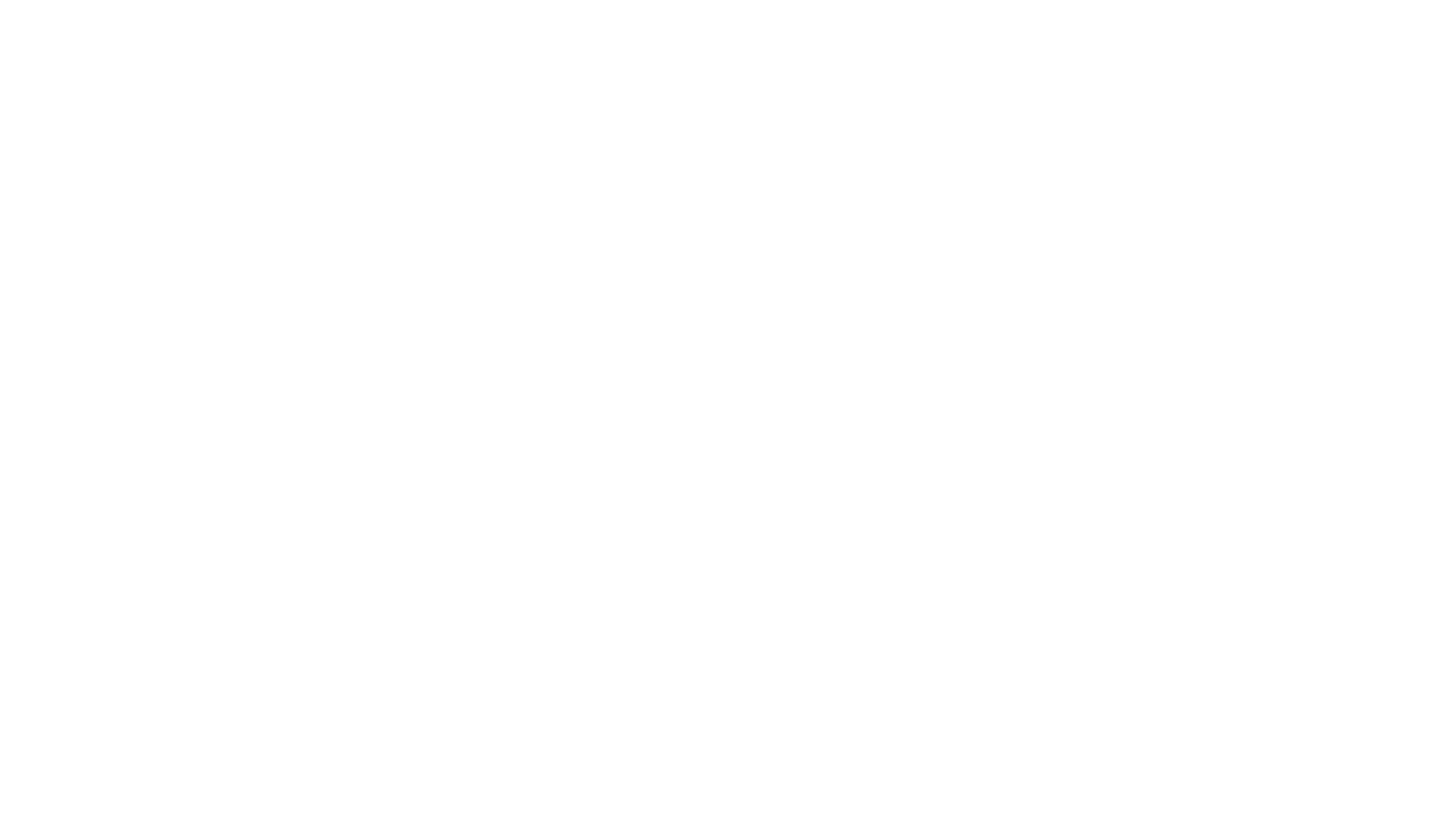

## Slide 7
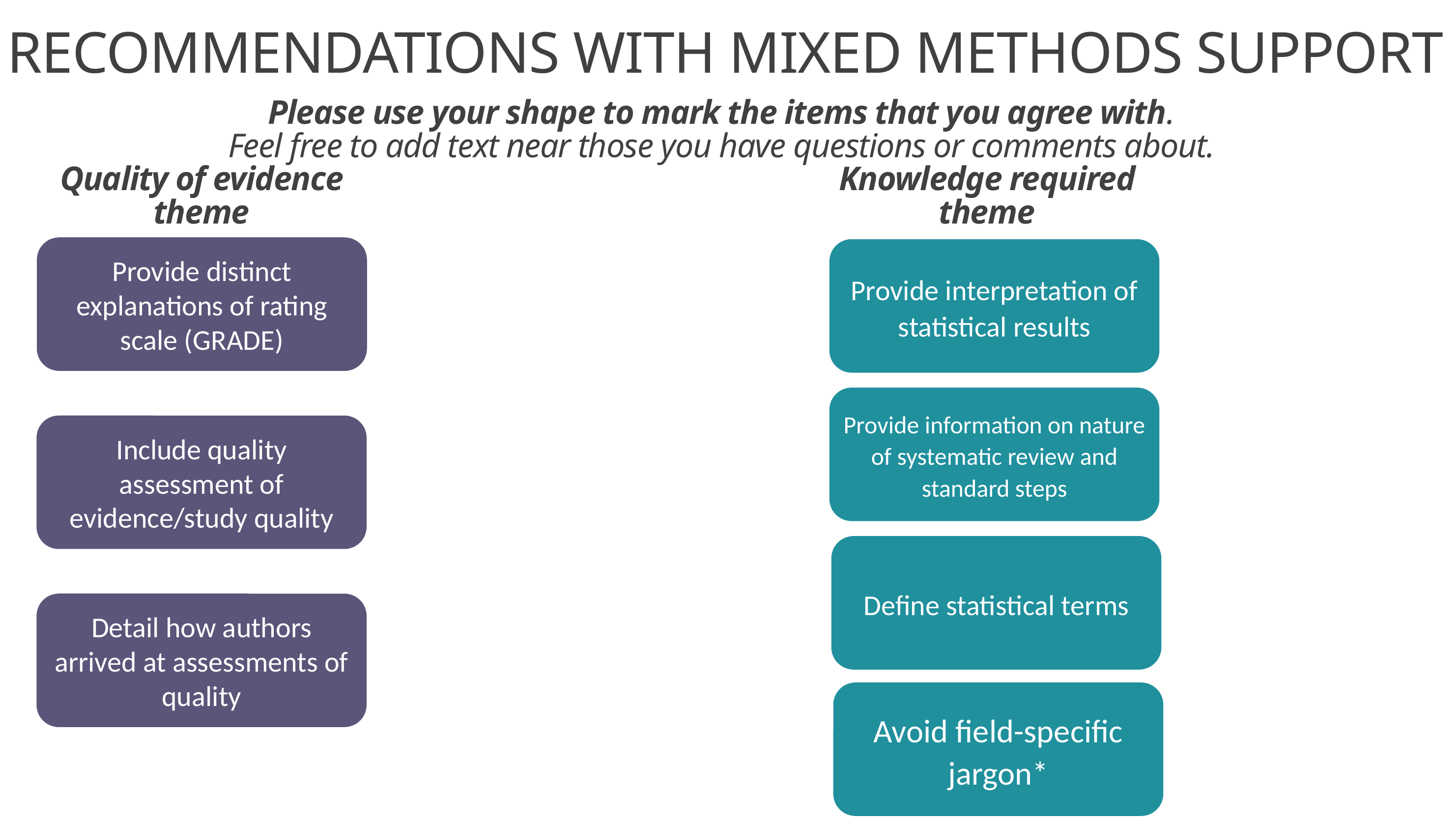

# RECOMMENDATIONS WITH MIXED METHODS SUPPORT
Please use your shape to mark the items that you agree with.
Feel free to add text near those you have questions or comments about.
Quality of evidence theme
Knowledge required theme
Provide distinct explanations of rating scale (GRADE)
Provide interpretation of statistical results
Provide information on nature of systematic review and standard steps
Include quality assessment of evidence/study quality
Define statistical terms
Detail how authors arrived at assessments of quality
Avoid field-specific jargon*

## Slide 8
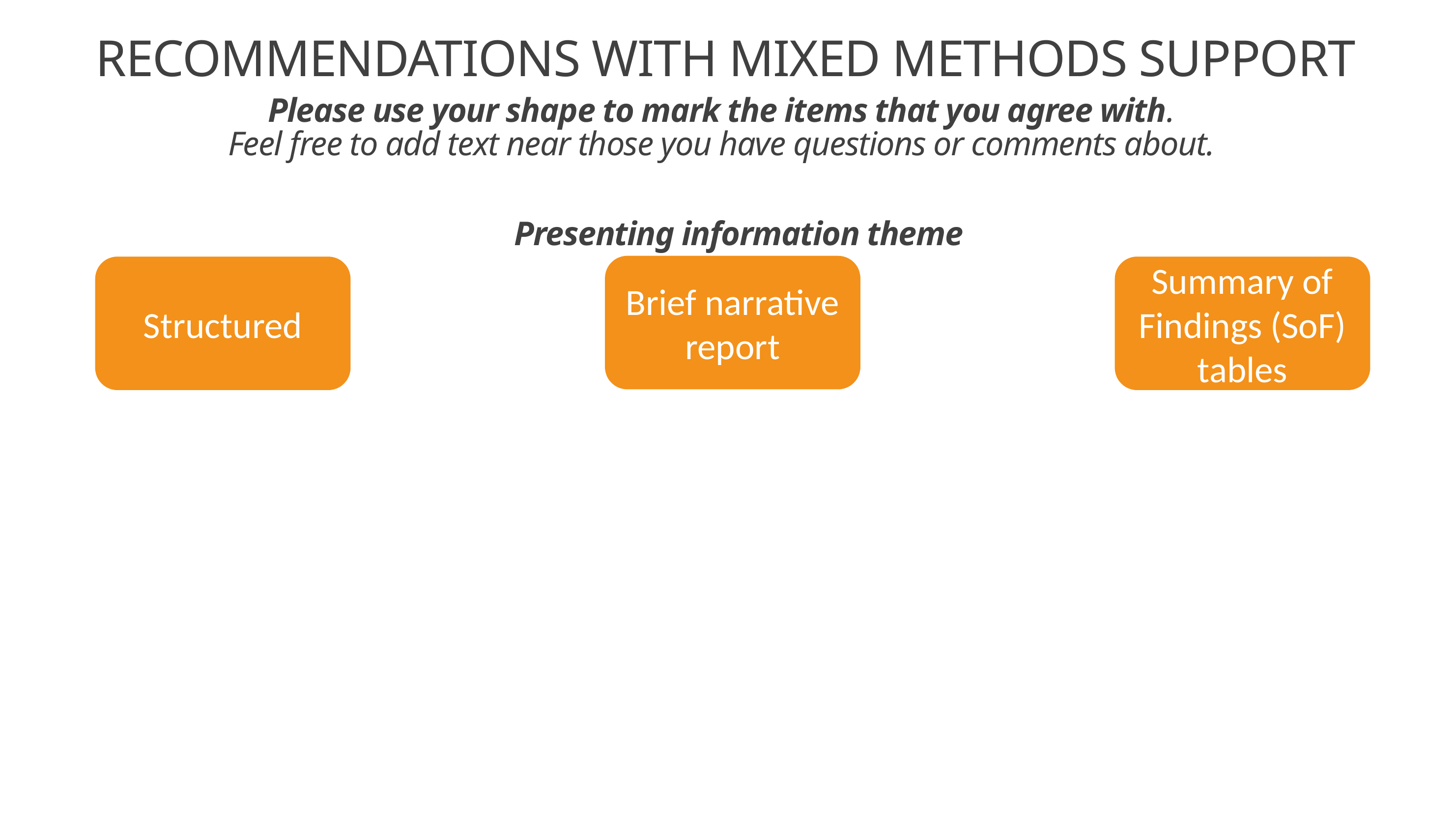

# RECOMMENDATIONS WITH MIXED METHODS SUPPORT
Please use your shape to mark the items that you agree with.
Feel free to add text near those you have questions or comments about.
Presenting information theme
Brief narrative report
Structured
Summary of Findings (SoF) tables

## Slide 9
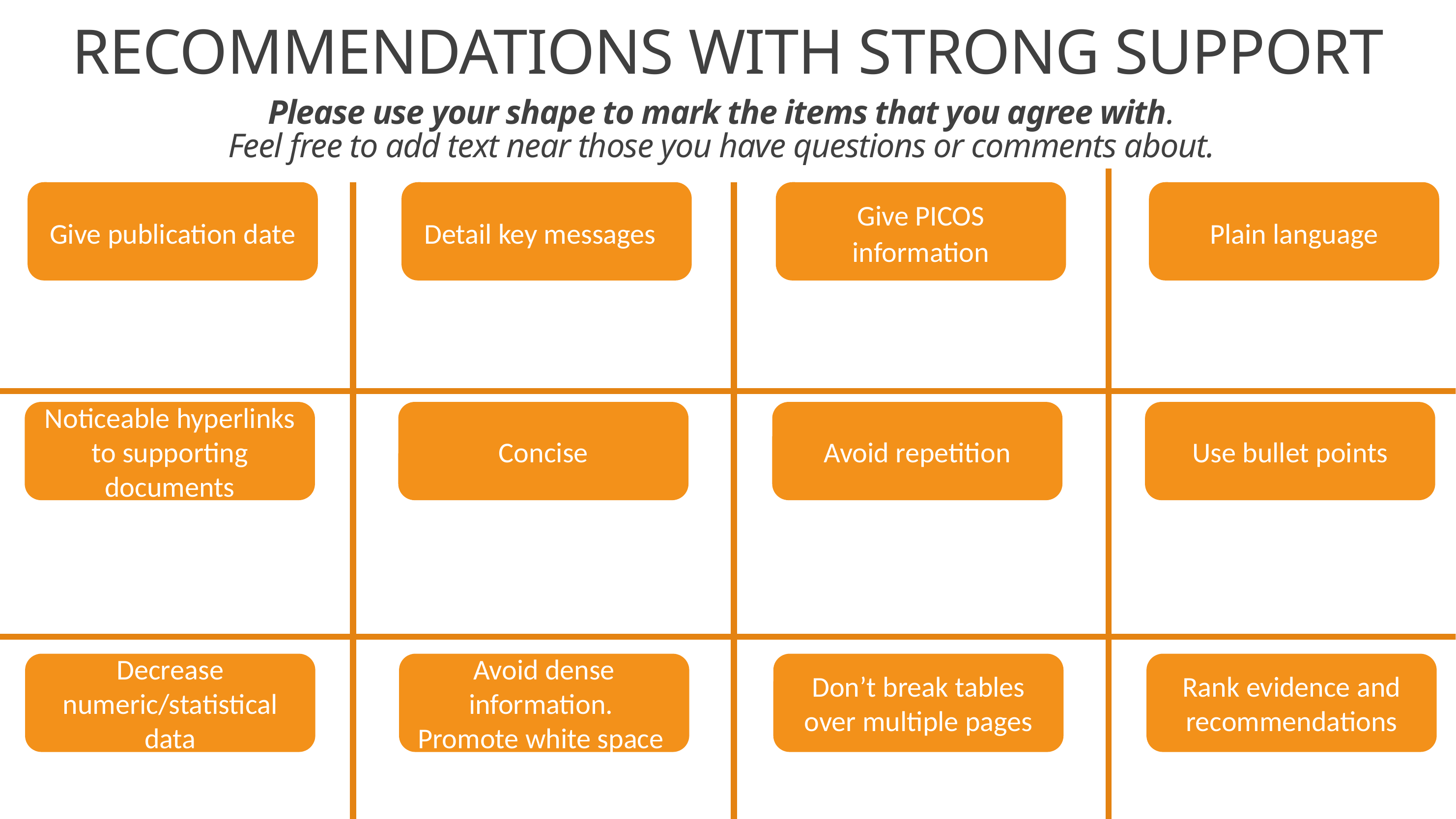

# RECOMMENDATIONS WITH STRONG SUPPORT
Please use your shape to mark the items that you agree with.
Feel free to add text near those you have questions or comments about.
Give publication date
Detail key messages
Give PICOS information
Plain language
Noticeable hyperlinks to supporting documents
Concise
Avoid repetition
Use bullet points
Decrease numeric/statistical data
Avoid dense information.
Promote white space
Don’t break tables over multiple pages
Rank evidence and recommendations

## Slide 10
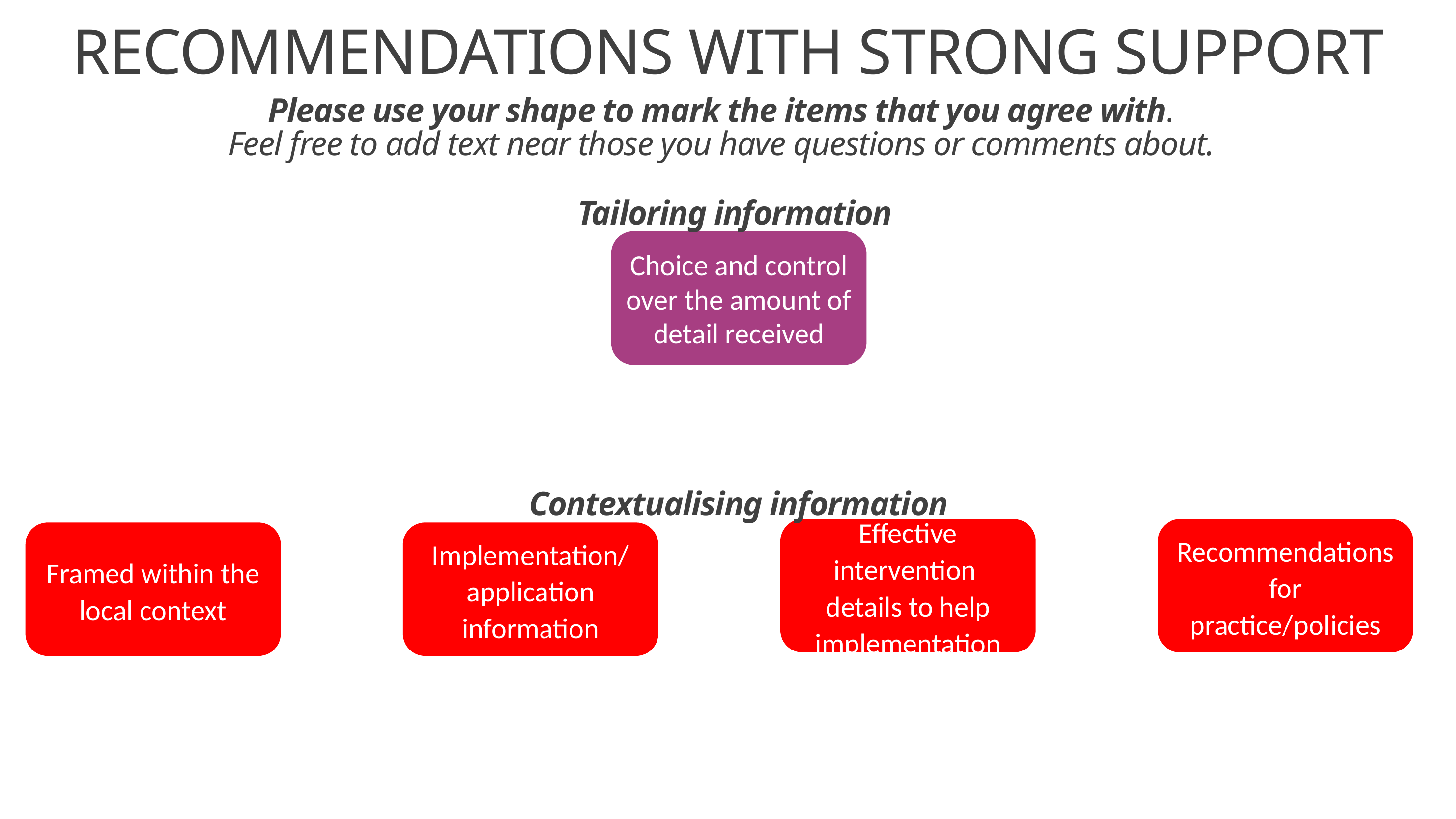

RECOMMENDATIONS WITH STRONG SUPPORT
Please use your shape to mark the items that you agree with.
Feel free to add text near those you have questions or comments about.
Tailoring information
Choice and control over the amount of detail received
Contextualising information
Effective intervention
details to help implementation
Recommendations for practice/policies
Framed within the local context
Implementation/ application information

## Slide 11
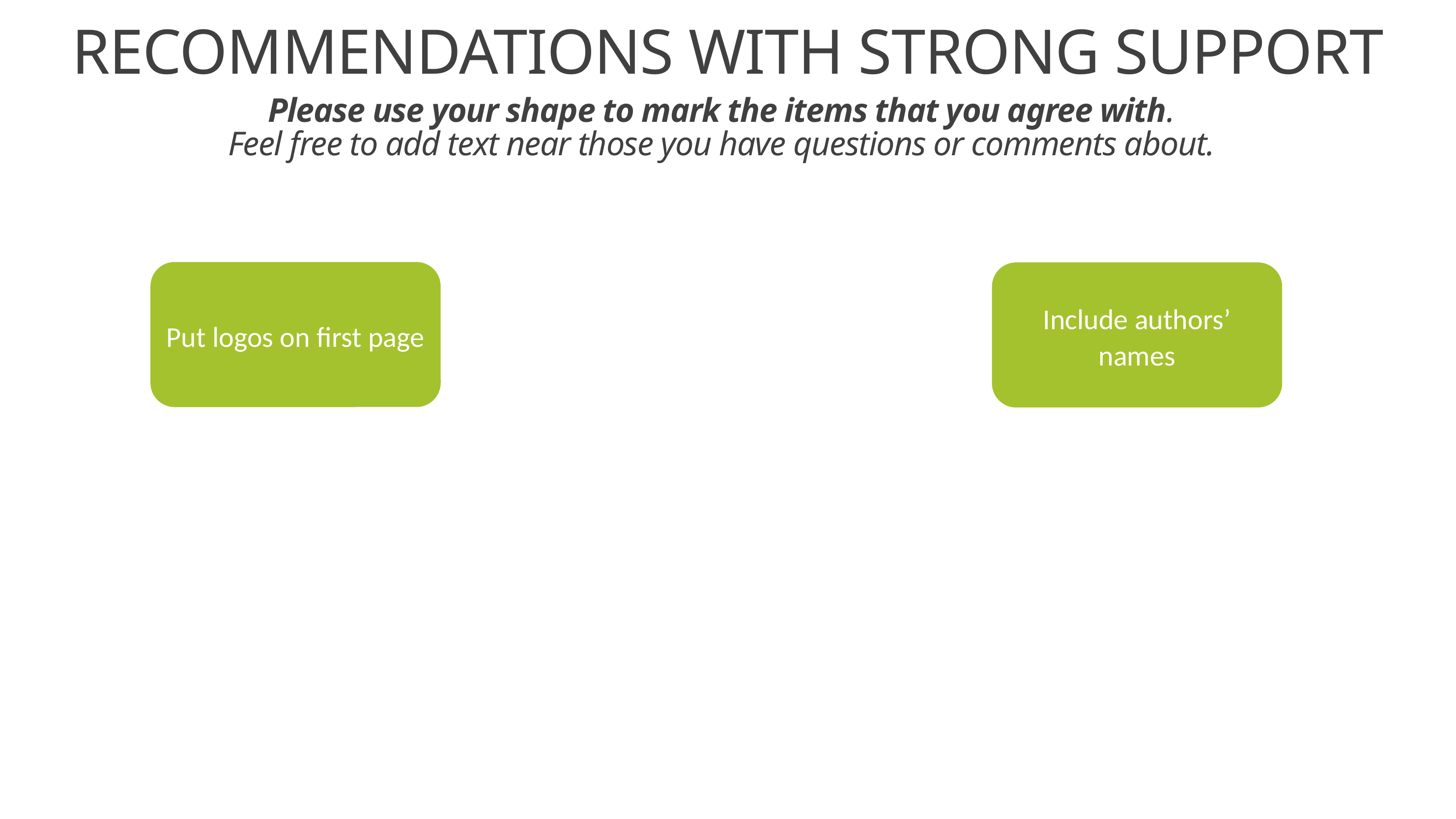

RECOMMENDATIONS WITH STRONG SUPPORT
Please use your shape to mark the items that you agree with.
Feel free to add text near those you have questions or comments about.
Put logos on first page
Include authors’ names

## Slide 12
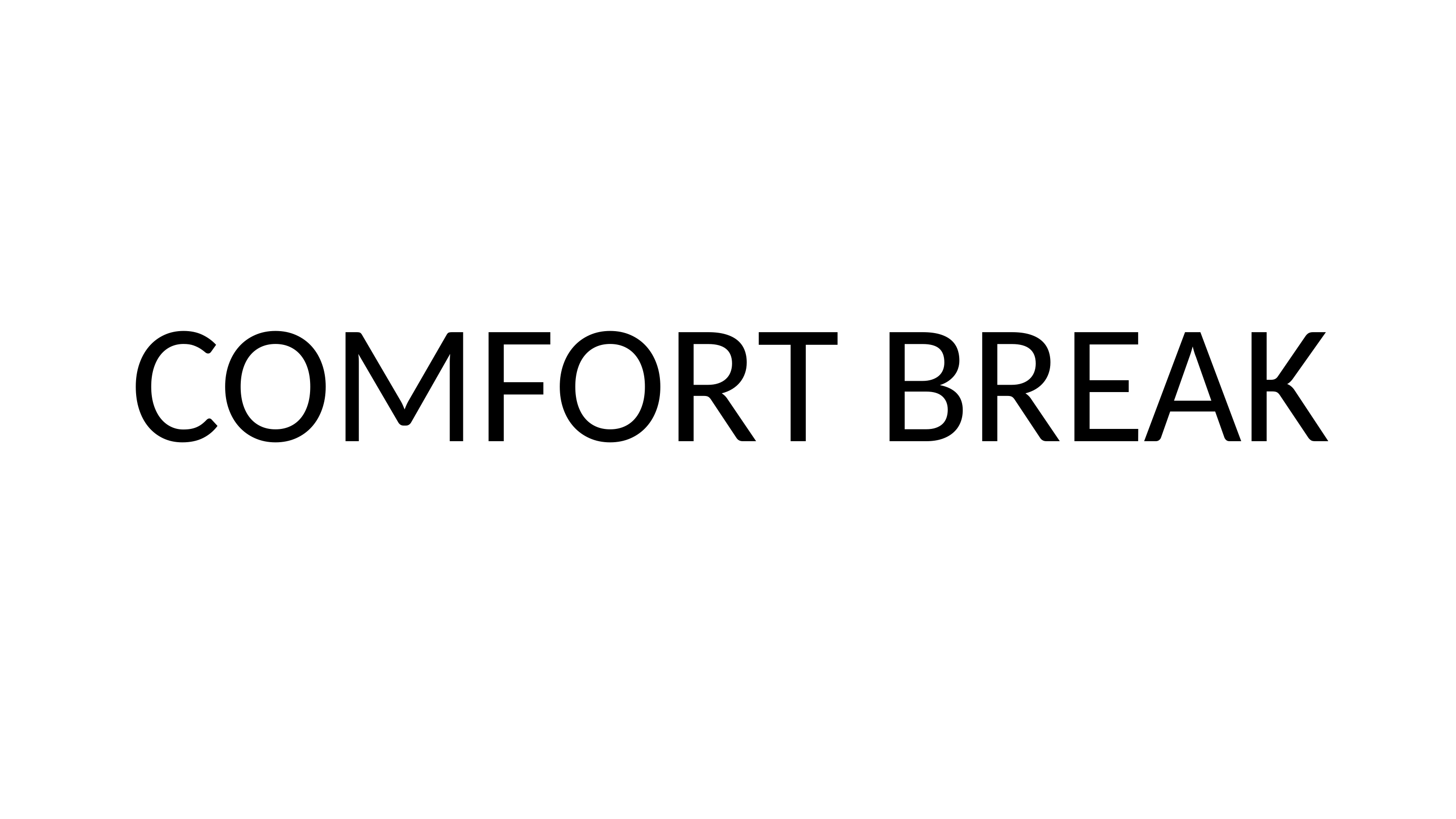

COMFORT BREAK

## Slide 13
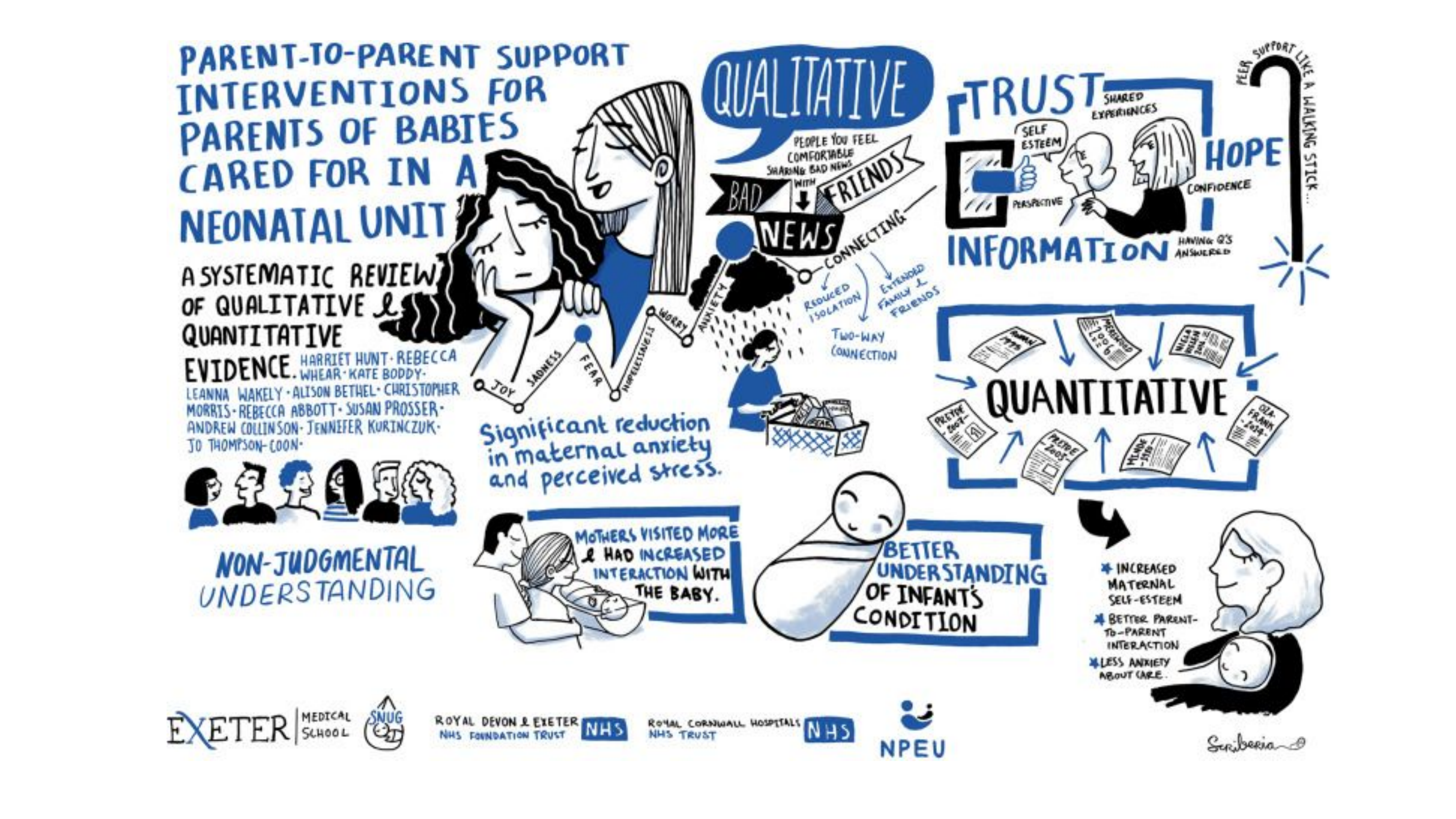

## Slide 14
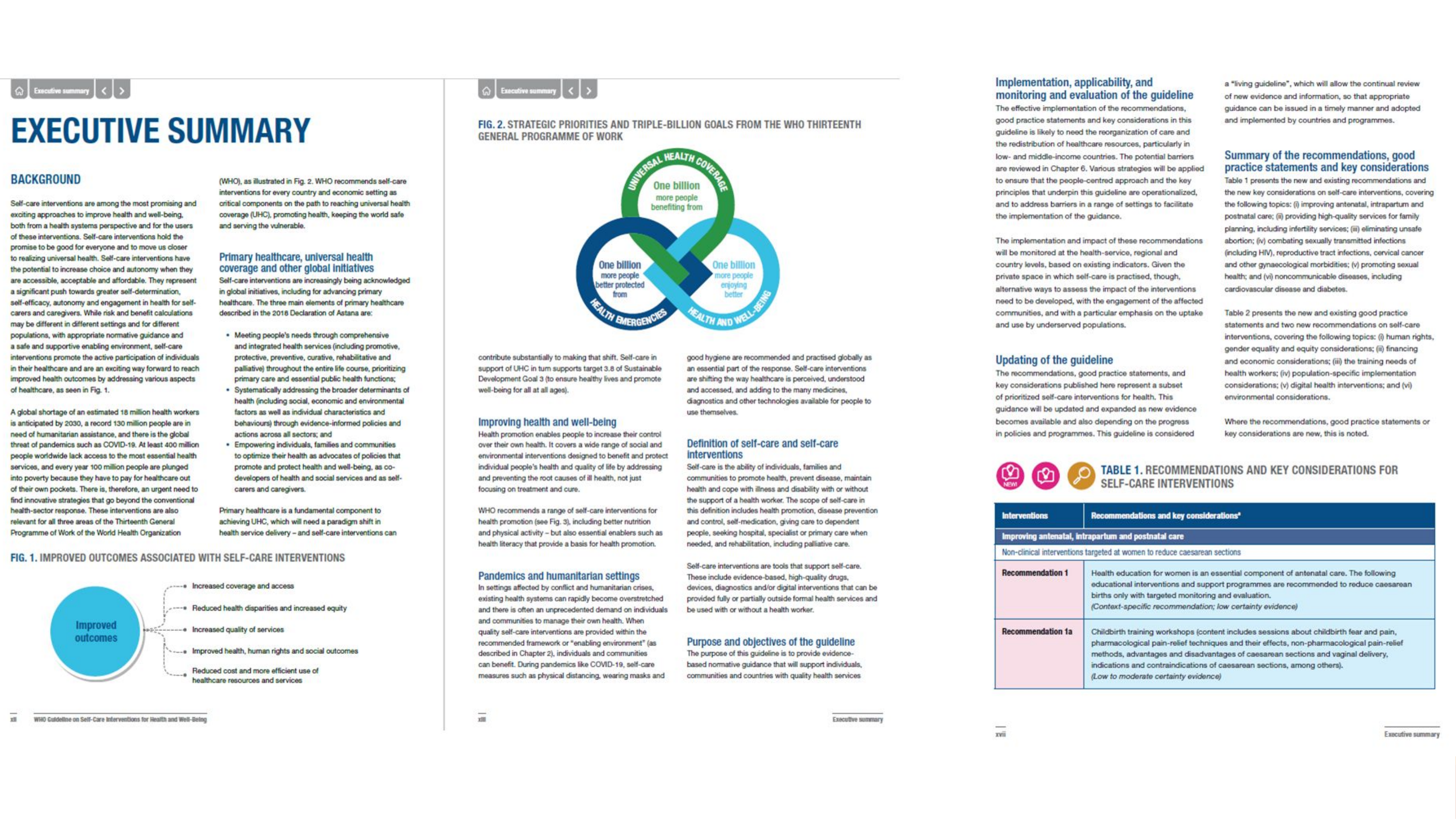

#

## Slide 15
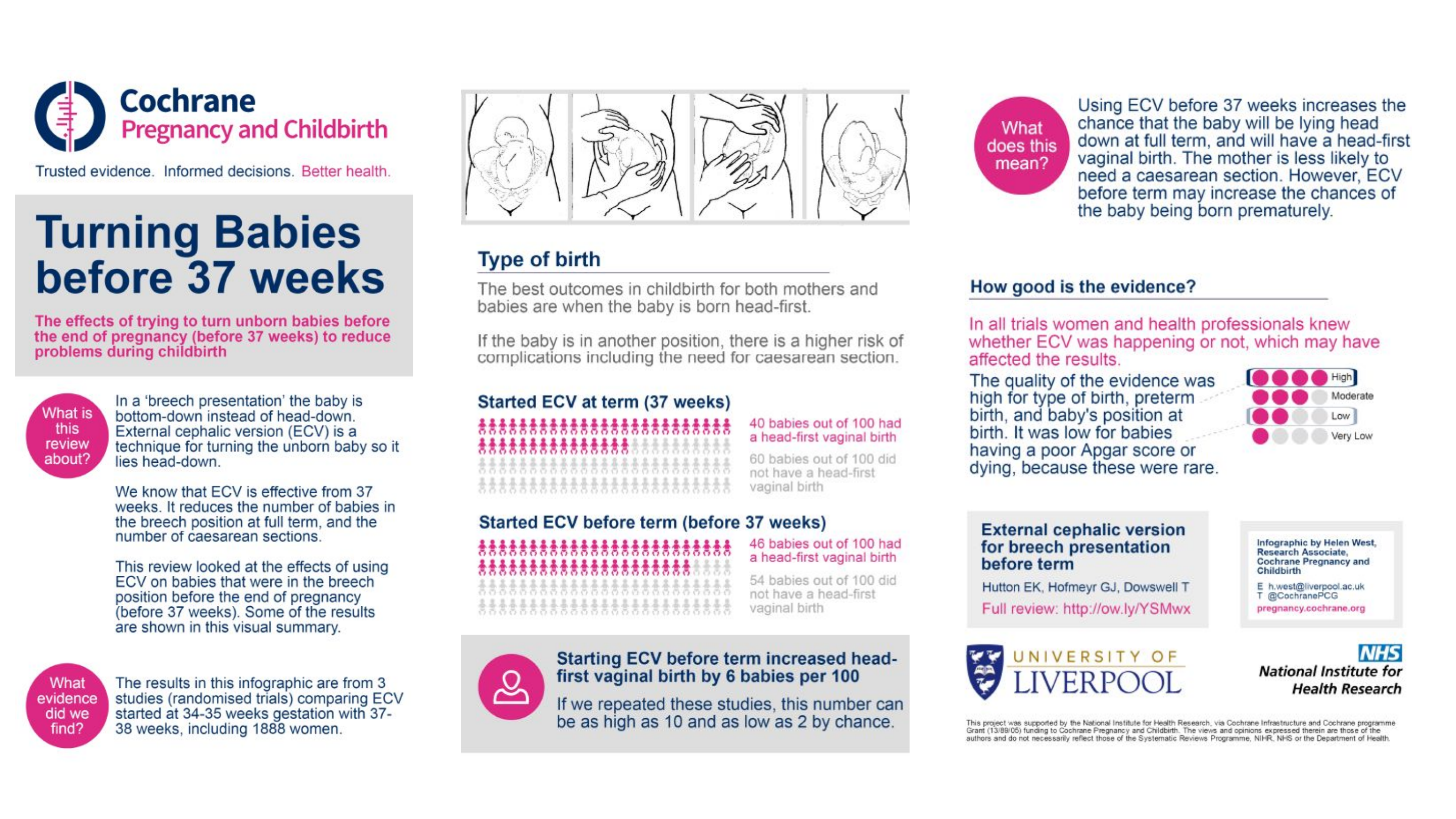

## Slide 16
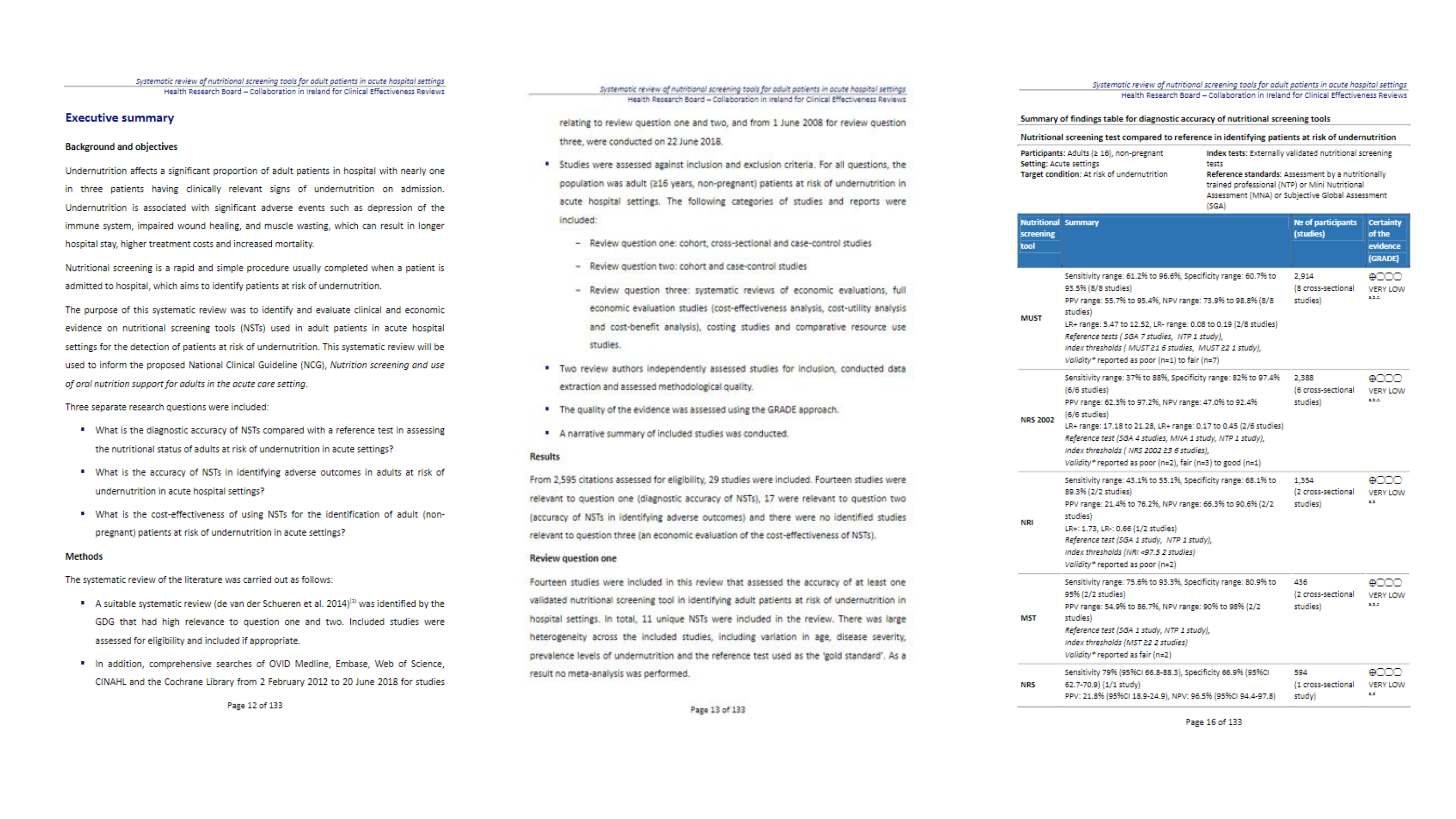

## Slide 17
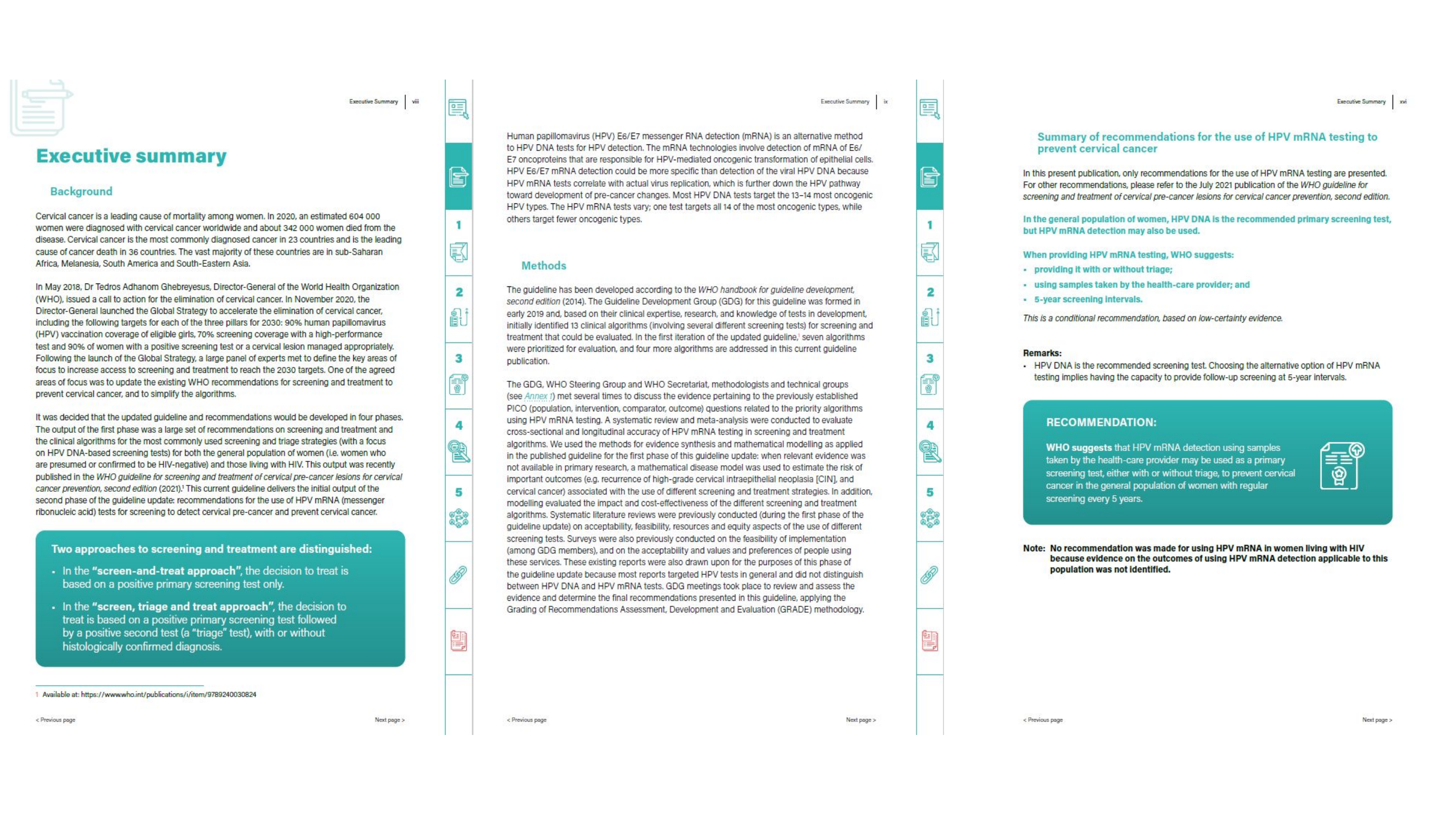

## Slide 18
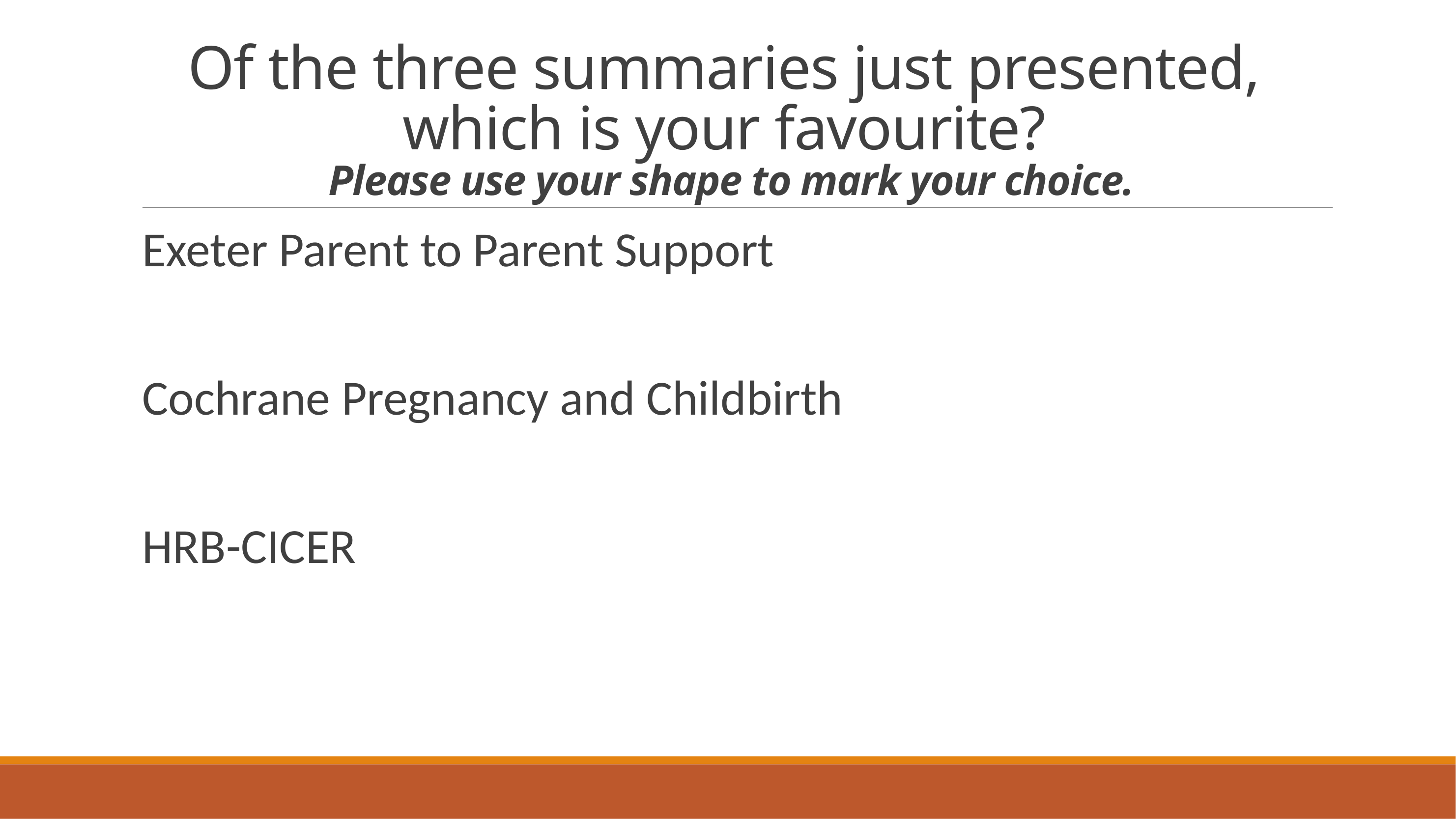

# Of the three summaries just presented, which is your favourite? Please use your shape to mark your choice.
Exeter Parent to Parent Support
Cochrane Pregnancy and Childbirth
HRB-CICER

## Slide 19
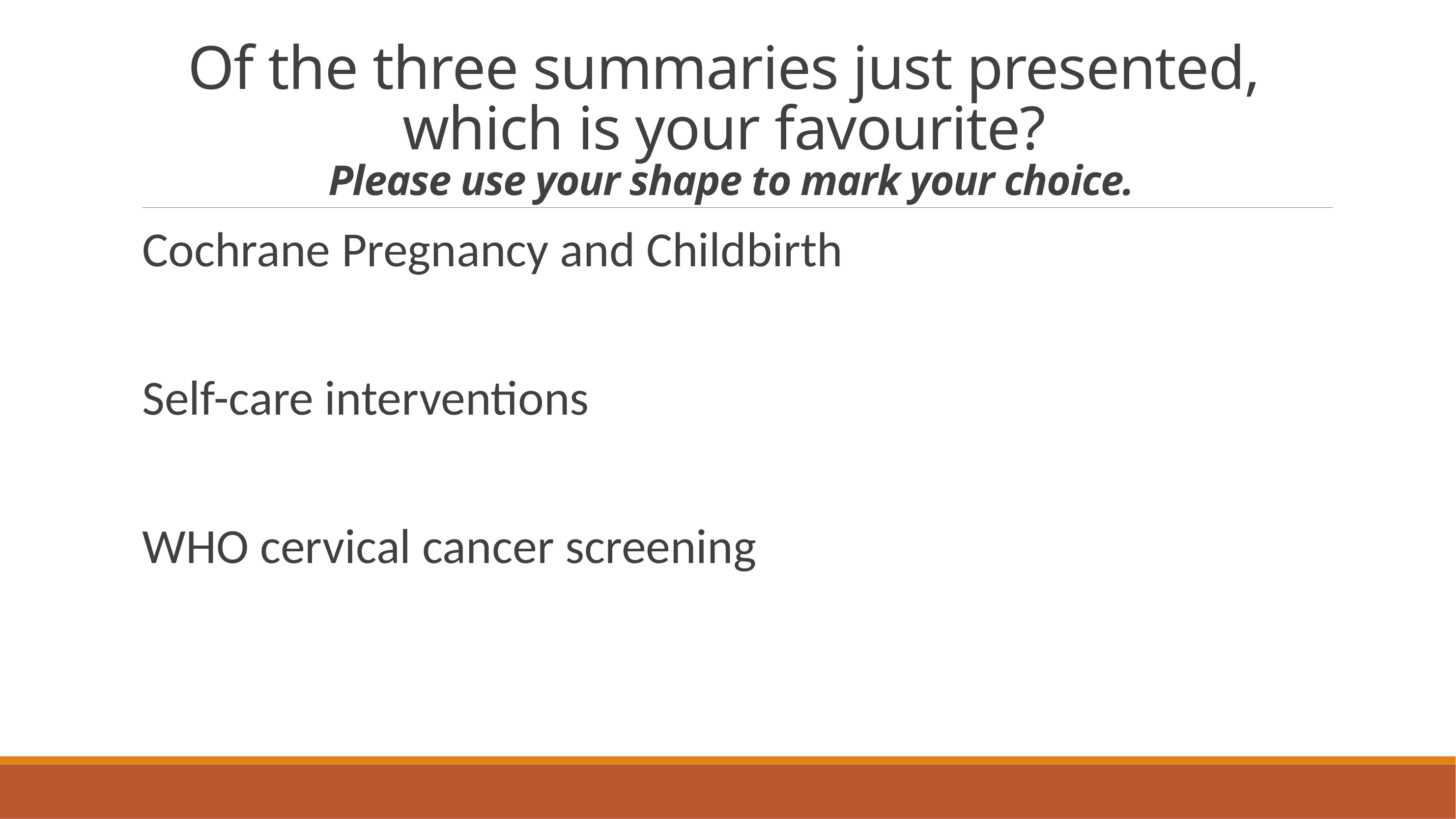

# Of the three summaries just presented, which is your favourite? Please use your shape to mark your choice.
Cochrane Pregnancy and Childbirth
Self-care interventions
WHO cervical cancer screening

## Slide 20
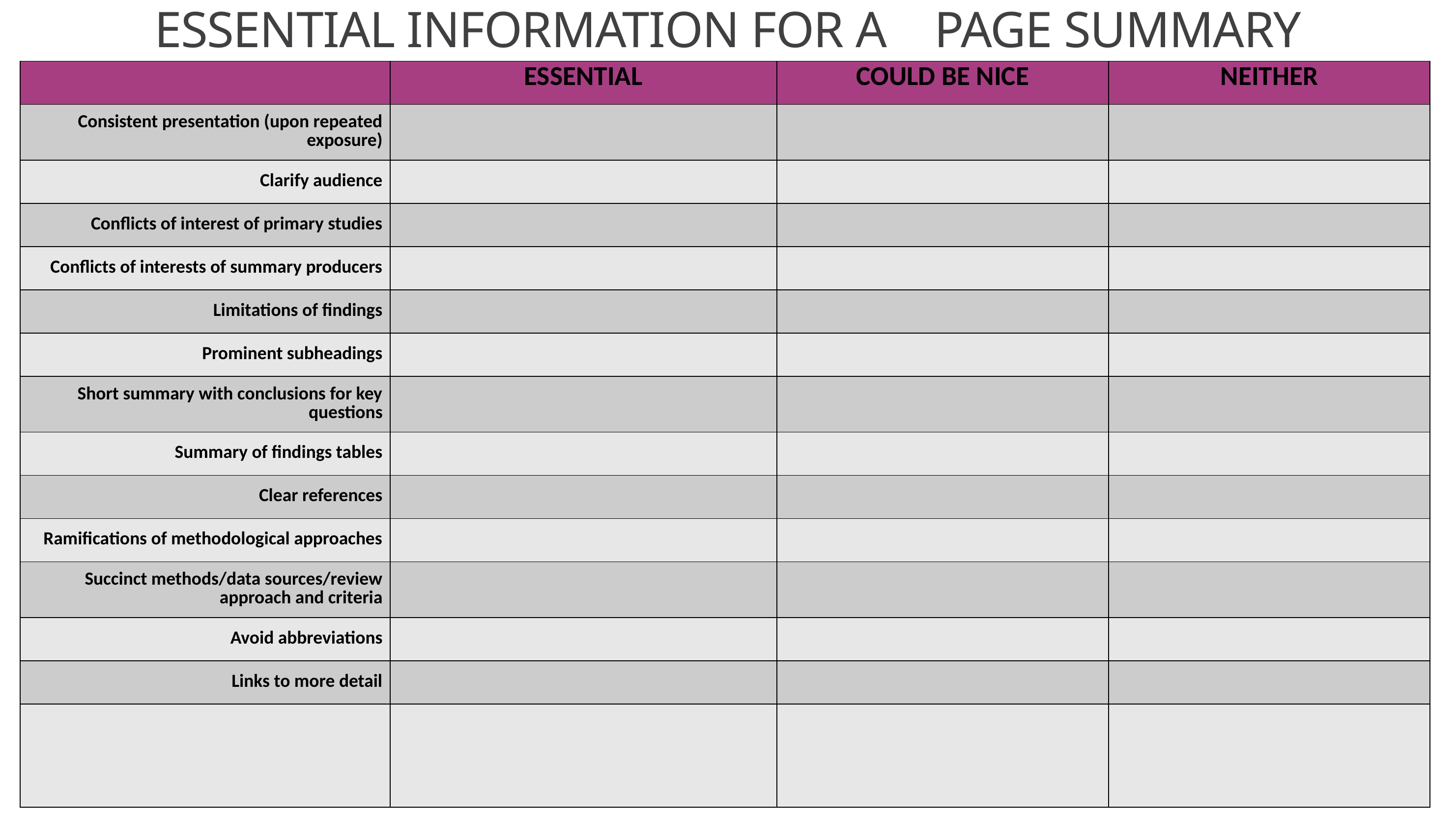

# ESSENTIAL INFORMATION FOR A    PAGE SUMMARY
| | ESSENTIAL | COULD BE NICE | NEITHER |
| --- | --- | --- | --- |
| Consistent presentation (upon repeated exposure) | | | |
| Clarify audience | | | |
| Conflicts of interest of primary studies | | | |
| Conflicts of interests of summary producers | | | |
| Limitations of findings | | | |
| Prominent subheadings | | | |
| Short summary with conclusions for key questions | | | |
| Summary of findings tables | | | |
| Clear references | | | |
| Ramifications of methodological approaches | | | |
| Succinct methods/data sources/review approach and criteria | | | |
| Avoid abbreviations | | | |
| Links to more detail | | | |
| | | | |
ESSENTIAL INFORMATION
COULD BE NICE
NEITHER

## Slide 21
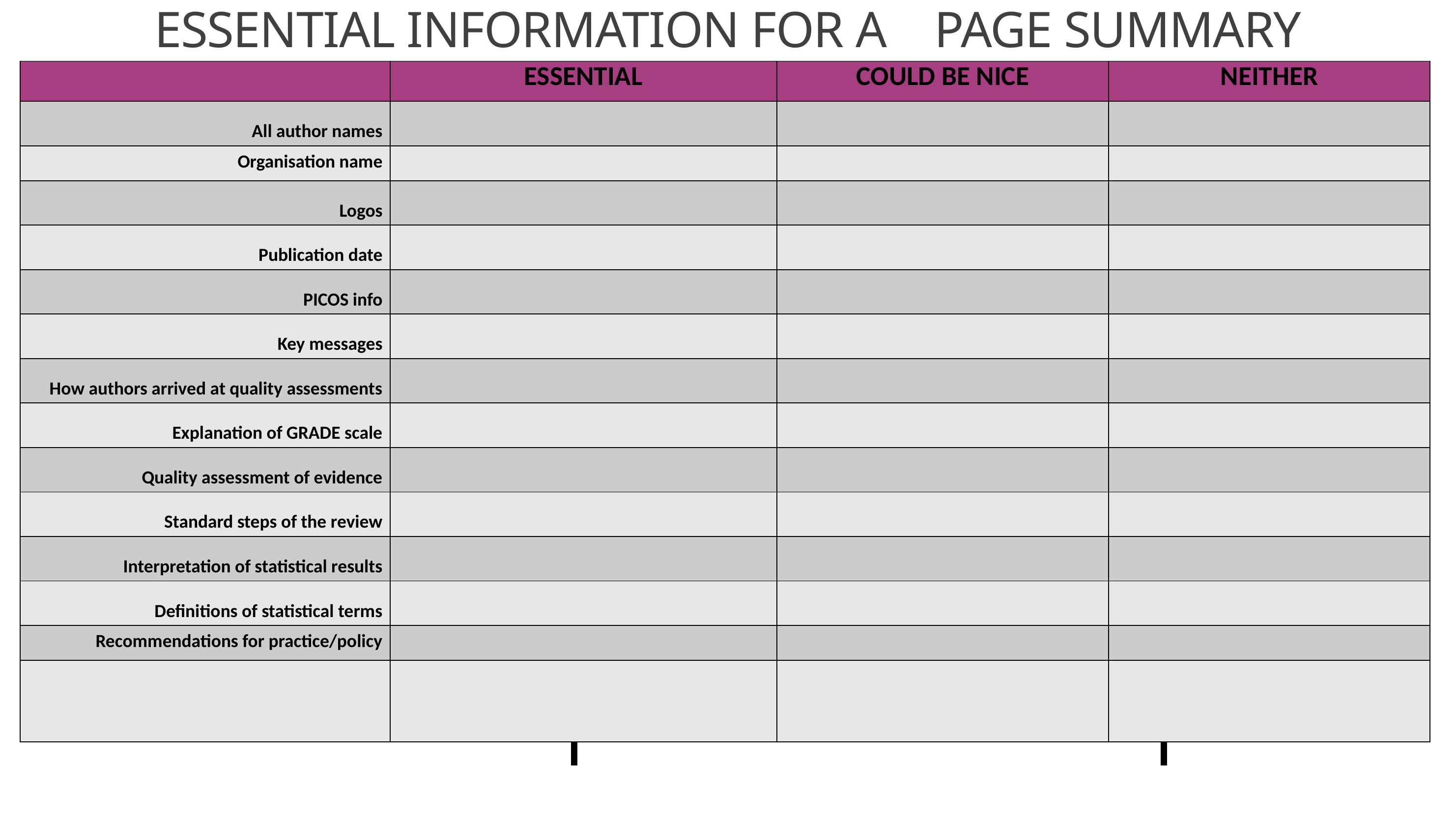

# ESSENTIAL INFORMATION FOR A    PAGE SUMMARY
| | ESSENTIAL | COULD BE NICE | NEITHER |
| --- | --- | --- | --- |
| All author names | | | |
| Organisation name | | | |
| Logos | | | |
| Publication date | | | |
| PICOS info | | | |
| Key messages | | | |
| How authors arrived at quality assessments | | | |
| Explanation of GRADE scale | | | |
| Quality assessment of evidence | | | |
| Standard steps of the review | | | |
| Interpretation of statistical results | | | |
| Definitions of statistical terms | | | |
| Recommendations for practice/policy | | | |
| | | | |
ESSENTIAL INFORMATION
COULD BE NICE
NEITHER

## Slide 22
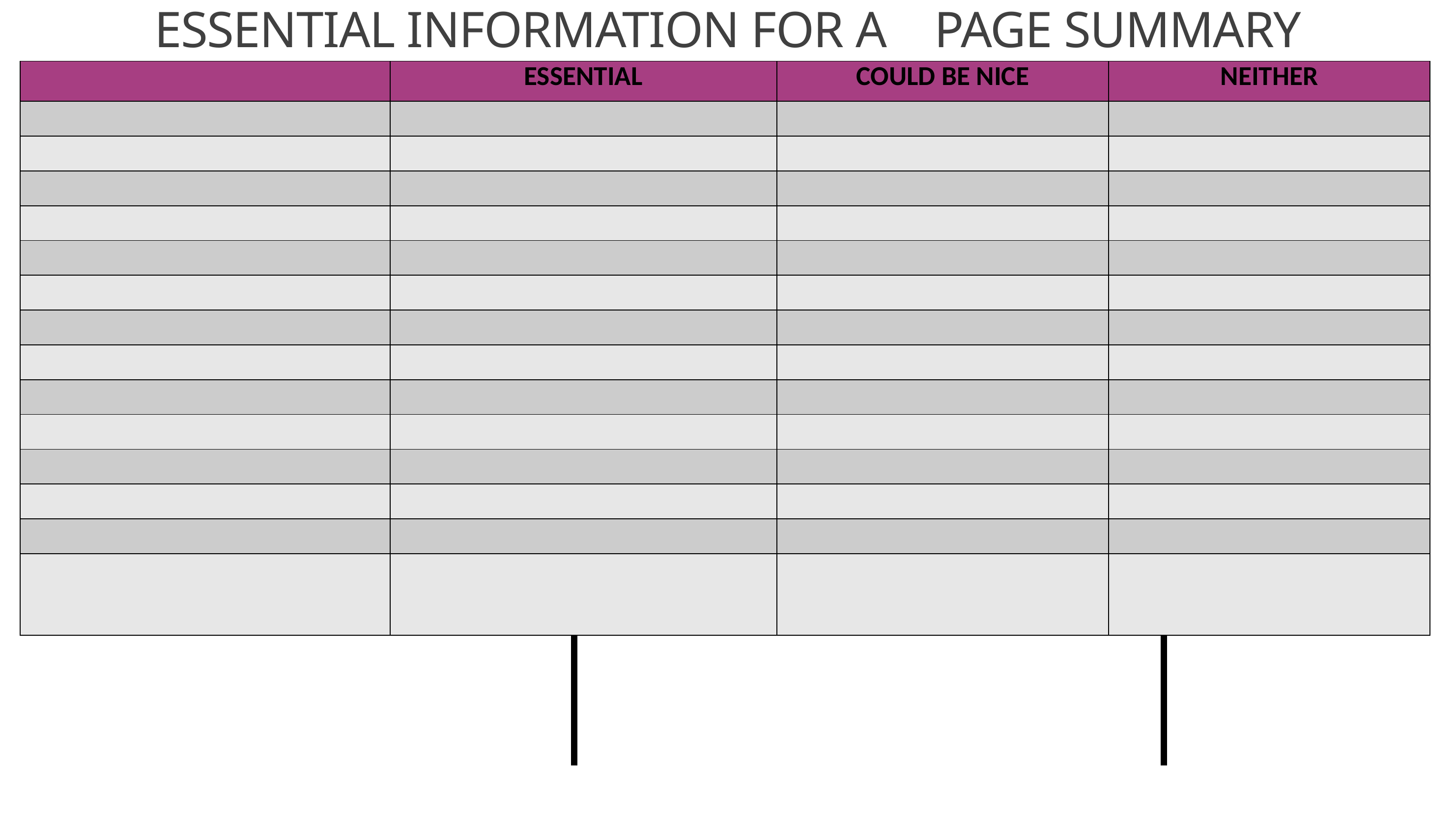

# ESSENTIAL INFORMATION FOR A    PAGE SUMMARY
| | ESSENTIAL | COULD BE NICE | NEITHER |
| --- | --- | --- | --- |
| | | | |
| | | | |
| | | | |
| | | | |
| | | | |
| | | | |
| | | | |
| | | | |
| | | | |
| | | | |
| | | | |
| | | | |
| | | | |
| | | | |
ESSENTIAL INFORMATION
COULD BE NICE
NEITHER
