# Supplementary material for: Co-design workshops to develop evidence synthesis summary formats for use by clinical guideline development groups
Source: Syst Rev. 2024 Mar 27;13:97. doi: 10.1186/s13643-024-02518-z (PMC10967093; doi:10.1186/s13643-024-02518-z)
Supplement: Supplementary file 5 — Additional file 5. Guidance for Summary Producers. [file 13643_2024_2518_MOESM5_ESM.docx]

**Appendix 5.** Evidence Summary Guidance

- Have you established your relationship with your commissioner and end-user as the evidence synthesis summary producer?
  - Clarify your audience as this may affect the need for more technical information, budget concerns, framing your findings, etc. in the summary
  - Check with the commissioner of the work about the appropriateness of providing recommendations (for care and policies) or ‘author’s conclusions’
- Does the reader know who produced the evidence synthesis summary?
  - Provide an organisation name, logo, or first author and a link or contact details available for the organisation or corresponding author
  - Provide a short conflict of interest statement (e.g., there are none or some) with link to further details in the full technical report
  - Be consistent across outputs in the order and format of how you present information
- Have you promoted accessibility for your reader?
  - Keep the summary as short as possible (1-2 pages)
  - Use visual formats (e.g., represent statistical data visually)
  - Embrace white space and balance imagery and text
  - Use structured formatting such as bullet points
  - Use prominent subheadings to chunk information into sections (Avoiding the IMRaD format.
  - Consider using questions as headings
  - If you have a summary of findings table, order the information by outcomes, time points or other relevant information to ‘chunk’ information
- Is your evidence synthesis summary interactive?
  - Provide hyperlinks to specific sections of the full technical report (e.g., conflicts of interest disclosures, glossary of statistical terms, methodology details, quality of evidence, results section); avoid footnotes
- Is your reader able to clearly tell what the key takeaway messages are?
  - Highlight key messages prominently (e.g., separated in its own coloured box)
- Does the reader know the scope of the contents of the evidence synthesis summary?
  - Briefly state why the review was done (scope and context of the work)
  - Describe the participants, interventions, comparators, and outcomes (PICO) in plain language
- Does the reader know how the evidence was gathered?
  - Provide a date indicating the recency of the information synthesised (e.g., publication *or* search date)
  - Provide review type (e.g., systematic, rapid, realist) and key restrictions affecting results
  - Avoid detailed methodological information (e.g., double screening or extraction, databases searched, etc.)
- Does the reader know about the overall quality of evidence of the body of evidence?
  - Briefly explain the quality of evidence scale (i.e., GRADE) in plain language or provide a link to further details in the full technical report
  - Include indication of the overall quality of evidence
